# Adsorbate chemical environment-based machine learning framework for heterogeneous catalysis

**DOI:** 10.1038/s41467-022-33256-2

**Published:** 2022-10-02

**Authors:** Pushkar G. Ghanekar, Siddharth Deshpande, Jeffrey Greeley

**Affiliations:** 1 Davidson School of Chemical Engineering, Purdue University, West Lafayette, IN 47907 USA; 2 Present Address: Department of Chemical Engineering, University of Delaware, Newark, DE USA

**Keywords:** Electrocatalysis, Surface chemistry, Computational methods, Heterogeneous catalysis

## Abstract

Heterogeneous catalytic reactions are influenced by a subtle interplay of atomic-scale factors, ranging from the catalysts’ local morphology to the presence of high adsorbate coverages. Describing such phenomena via computational models requires generation and analysis of a large space of atomic configurations. To address this challenge, we present Adsorbate Chemical Environment-based Graph Convolution Neural Network (ACE-GCN), a screening workflow that accounts for atomistic configurations comprising diverse adsorbates, binding locations, coordination environments, and substrate morphologies. Using this workflow, we develop catalyst surface models for two illustrative systems: (i) NO adsorbed on a Pt_3_Sn(111) alloy surface, of interest for nitrate electroreduction processes, where high adsorbate coverages combined with low symmetry of the alloy substrate produce a large configurational space, and (ii) OH* adsorbed on a stepped Pt(221) facet, of relevance to the Oxygen Reduction Reaction, where configurational complexity results from the presence of irregular crystal surfaces, high adsorbate coverages, and directionally-dependent adsorbate-adsorbate interactions. In both cases, the ACE-GCN model, trained on a fraction (~10%) of the total DFT-relaxed configurations, successfully describes trends in the relative stabilities of *unrelaxed* atomic configurations sampled from a large configurational space. This approach is expected to accelerate development of rigorous descriptions of catalyst surfaces under in-situ conditions.

## Abstract (web summary)

A combination of electronic structure calculations and machine learning strategies is developed to predict structures of complex heterogeneous catalysts in realistic environments, yielding new opportunities for optimization for energy applications.

## Introduction

Theoretical computational models have become indispensable in elucidating the intricate molecular-level details of heterogeneous catalysts. High-throughput material screening strategies, combined with descriptor-based correlations such as scaling and Brønsted–Evan–Polanyi relationships^1–4^, have played a central role in identifying promising candidates for important oxygen, nitrogen, and carbon-based chemistries. These approaches have been augmented by the recent emergence of improved computational modeling algorithms, some based on machine learning, which have made screening of diverse materials classes, including oxides, perovskites, zeolites, and metal-organic frameworks (MOFs), possible through the facile generation of diverse materials-specific structural motifs^5–10^. Accelerated predictions of binding energies of reaction intermediates have further contributed to the descriptor-based catalyst screening paradigm^6,7,11–15^. These computational strategies, which iteratively improve through experience, have enabled the (re)discovery of exciting catalytic materials and chemical insights. In spite of these advances, however, it remains challenging to predict the exact nature of heterogeneous catalyst active sites under reaction conditions, as the catalyst properties are highly sensitive to the atomic-scale complexities arising from adsorbate-adsorbate interactions, the local morphology of the catalysts, and environmentally induced variations in the catalyst’s surface composition^16–22^. To successfully overcome these difficulties, efficient generation and analysis of atomistic models is critical and requires development of methods that can efficiently sample the large configurational space of surface atomic configurations resulting from diverse catalyst compositions and surface structures^23,24^.

Motivated by the above challenges, a variety of studies have developed machine learning-based surrogate models to predict the adsorption energies of model adsorbates on low-index surface facets of metal and oxide catalysts. However, although these studies have contributed important insights, the proposed workflows are generally customized for the examples being studied or for user-curated datasets comprised of a particular binding site or a particular unit cell geometry. To permit the extension of machine learning-based approaches to catalytic systems where one, or more, of the complex phenomena described above are relevant, we propose a generalized screening workflow that can describe diverse chemical environments, independent of the shape and size of the unit cell, the nature and number of binding sites, and the identity of the adsorbates. Such a flexible framework, which is described in more detail below, has not yet, to the best of our knowledge, been proposed for studying adsorbate interactions with a catalyst surface^6,13,15,25–31^.

The proposed approach involves systematic enumeration of atomic configurations using graph-based representations^23^. The relevant chemical and geometric properties of the generated motifs are then learned and mapped to the target property of choice using a machine learning model based on a graph neural network architecture^32,33^, which is termed the Adsorbate Chemical Environment-based Graph Convolution Neural Network (ACE-GCN). One of the key distinguishing factors of the ACE-GCN algorithm is the utilization of subgraphs, which are flexible and powerful representations for probing adsorption energetics of high coverages of adsorbates in structurally diverse heterogeneous catalytic systems. Among other benefits, the use of subgraphs leads to a compact representation of surface environments, leading to enhanced efficiency that may elude full graph representations. ACE-GCN serves as a surrogate model for expensive electronic-structure optimization routines and efficiently provides estimates for the target properties of catalyst surfaces, thereby facilitating high throughput evaluation of a large space of complex surface site models.

This ability is demonstrated in the context of two catalytic systems that are relevant to practical electrocatalytic applications and that represent the typical complexities encountered when developing computational models of heterogeneous catalysts. The first case treats high coverage configurations of the adsorbate NO* on a Pt_3_Sn(111) terrace surface, wherein a vast surface configurational space resulting from both the reduction in the catalyst surface symmetry due to alloying^34–37^ and the strong binding nature of NO* yields rich catalytic behavior. This chemistry is of interest in electrocatalytic water treatment strategies, and similar complexities arise in chemistries such as Fischer-Tropsch synthesis and water-gas shift^17,38^. With our proposed workflow, all high coverage NO* configurations (~3400) are analyzed by performing a small fraction of DFT calculations (~350). In the second case, the challenge of modeling irregular or defected crystal surfaces, together with strong, directionally dependent adsorbate-adsorbate interactions, is addressed. High coverage configurations of OH*, known to be stabilized through intermolecular hydrogen bonds (H-bonding), are analyzed on the Pt(221) stepped and Pt(100) square surfaces. These types of interactions can strongly impact the energetics of electrocatalytic reactions such as hydrogen evolution, oxygen reduction, and CO electro-oxidation^39–42^. An approach inspired by transfer learning is employed, wherein explicit DFT calculations of high coverage OH* configurations on Pt(100) terraces (~200) are combined with selected calculations of OH* on Pt(221) (~400). Using the ACE-GCN approach, and subsequently including a modest number of additional high coverage geometries (~800) for incremental model improvement, a comprehensive set of high coverage OH* configurations on the Pt(221) surface (~11500) is explored to identify low energy adsorbate structures. This generalized approach shows how multiple datasets may be used to incorporate information from diverse catalyst morphologies to efficiently describe complex, low symmetry surfaces with large configurational spaces in the ACE-GCN framework^43–45^, and we expect that these techniques could, ultimately, be extended to treat additional complexities, including the presence of multidentate adsorbates and non-metallic frameworks such as zeolites and ionic solids^23^. We close by briefly illustrating the utility of these approaches for determining realistic, in-situ catalyst structures by analyzing the state of the Pt(221) surface under electrochemical conditions via an ab initio Pourbaix analysis.

## Results and discussion

Prediction of catalyst structures under realistic reaction conditions requires addressing two primary sources of complexity: (i) the structural intricacies of the catalyst, stemming from variations in compositional and morphological properties, and (ii) adsorbate structures, which may involve multiple adsorbed species and directionally dependent adsorbate-adsorbate interactions such as hydrogen bonding. These chemical complexities yield a large phase space of possible atomic configurations, motivating the development of a systematic computational framework to screen configurations with less expense than is required by exhaustive first principles analysis.

### Workflow and ACE-GCN framework

**Fig. 1:**
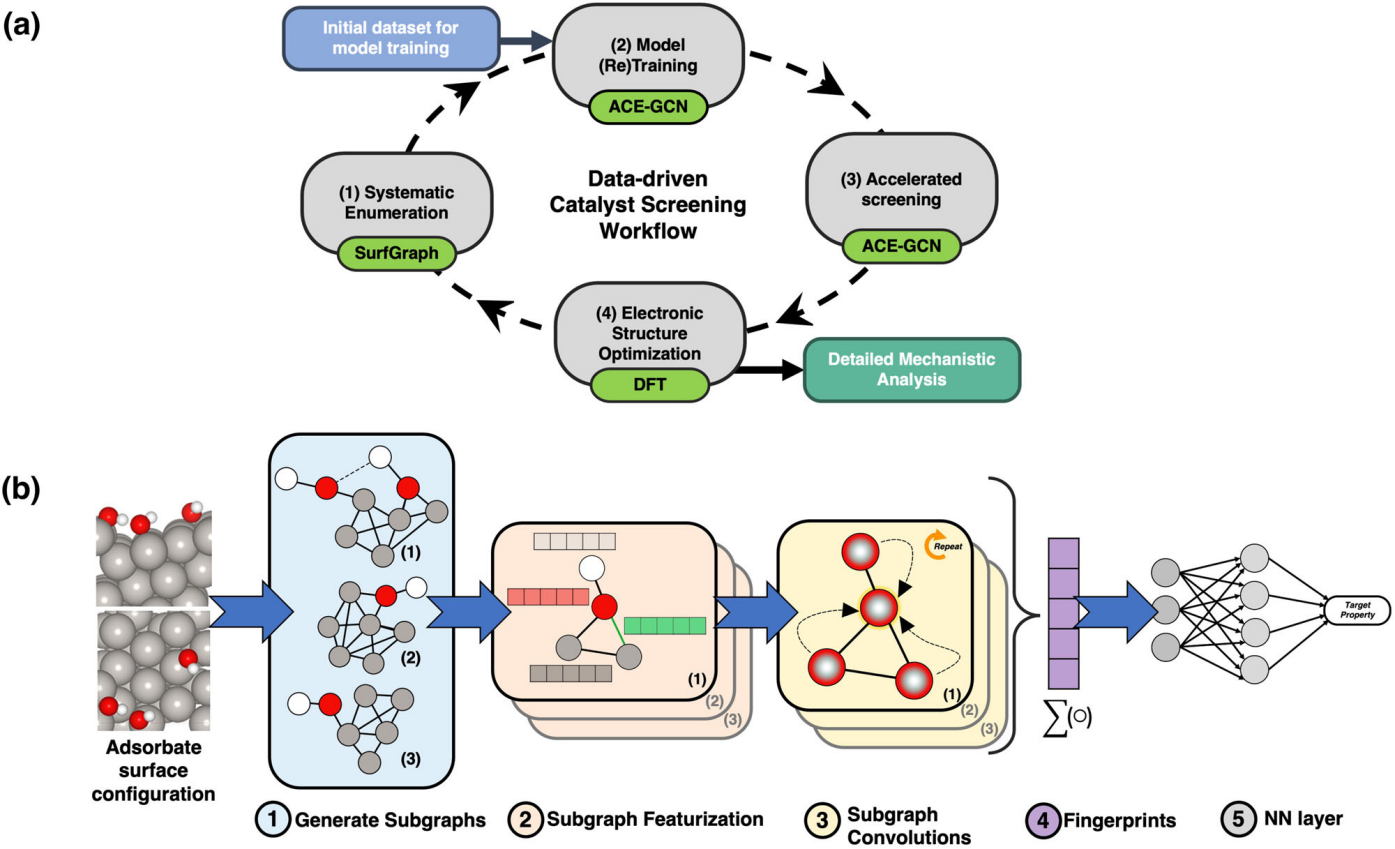
Catalyst screening workflow and overview of the ACE-GCN algorithm. **a** Screening workflow for identifying stable surface adsorbate configurations. The workflow demonstrates an incremental training approach to predict thermodynamically stable catalytic configurations. The cyclic workflow includes the following steps: (1) Systematic enumeration: all possible and unique high coverage surface adsorbate representations are generated using the SurfGraph algorithm, (2) Model Training:  the ACE-GCN model is (re)trained on selected structures utilizing the relevant surface representations identified in the previous steps, (3) Accelerated screening: the unrelaxed surface configurations generated in step 1 are ranked using the ACE-GCN model, which is pre-trained on a smaller subset of relevant DFT-relaxed case, and (4) Electronic structure optimization: selected *unrelaxed* configurations ranked by ACE-GCN are optimized using an electronic structure optimization code of choice and then utilized either for subsequent analysis or to re-train and improve the ACE-GCN model. **b** ACE-GCN algorithm to encode and train high coverage adsorbate configurations. (1) Generate subgraphs: each configuration is split into multiple subgraphs as identified by the SurfGraph algorithm. A distinct ego-graph is generated for each adsorbate to encode local geometric and chemical properties around the adsorbate in a subgraph representation, (2) Subgraph featurization: each atom and its corresponding bond attribute in the subgraph is expressed as a vector representation according to the chemical identity (elemental properties) and spatial bond distance, termed as node and edge features, respectively, (3) Subgraph convolutions: every node vector in the subgraph is iteratively updated through multiple rounds of graph convolution operations, which account for the atom’s geometric and chemical neighborhood using node and edge vectors of the neighboring atoms, (4) Fingerprints: a hierarchical pooling operation condenses all subgraphs for every adsorbate into one fingerprint vector, (5) NN layer: the fingerprint vector is passed to a feed-forward neural network (NN) which maps it to the target property of choice, such as the average adsorption energy.

Figure 1(a) summarizes the proposed screening framework. The cyclic workflow is divided into four parts: (i) systematic enumeration of unique atomic configurations, (ii) (re)training of the surrogate model with data of incremental complexity, (iii) accelerated screening using the surrogate model to identify the most relevant configurations amongst possible geometries, and (iv) electronic-structure relaxation of selected structures, which can be used for in-depth mechanistic analysis or to improve the surrogate model.

First, adsorbate configurations are generated by enumerating adsorbate binding locations on the catalyst surface using the SurfGraph algorithm^23^. This algorithm utilizes graph-based representations to identify and create unique surface adsorbate configurations, systematically accelerating the task of generating complex catalytic model motifs^23,24^. Next, ACE-GCN is utilized as a surrogate model for screening the generated motifs. The algorithm captures the geometric and chemical properties of a given surface adsorbate’s local environment and maps them to a target property of choice. In this work, ACE-GCN is initially trained on a small subset of relaxed adsorbate configurations, and then utilized as a surrogate model to systematically rank the energies of a much larger number of *unrelaxed* adsorbate configurations. The approach thus provides a framework to efficiently identify a subset of highly promising candidate structures, as generated by SurfGraph, for subsequent electronic-structure relaxation, therefore bypassing the computationally expensive step of DFT analysis of all possible atomistic configurations. After electronic structure optimization of the most promising structures, the selected candidate configurations are used to further improve the prediction capabilities of the ACE-GCN model by including them in an expanded training pool. Ultimately, the resulting surrogate model can be applied in various ways, including in-depth analysis of reaction mechanisms, evaluation of adsorbate partition functions and associated configurational entropy effects, or even training of user-defined Hamiltonians, such as cluster expansions, for specific areas of application^29,46,47^. Below, additional descriptions of the ACE-GCN framework, as well as two examples of its application, are provided.

### Adsorbate chemical environment-based graph neural networks

The ACE-GCN framework is based on a graph neural network (GNN) architecture^32,48^. Graph-based learning, wherein small molecules or crystals are presented as undirected graphs with atoms described as nodes and edges representing the connections between the atoms, has been used to accurately account for the underlying structural and chemical properties of a diverse class of materials, including small molecules^48^, periodic materials^32,49^, metal-organic frameworks^8^, and selected surfaces^6^. However, as discussed above, a successful implementation of such graph-based representations for complex surface models, incorporating a combination of multiple adsorbates, high coverage ensembles, and complex surface geometries (steps, kinks, and other defects), remains highly challenging. The ACE-GCN model constitutes a simple strategy for treating these sources of complexity.

The schematic in Fig. 1b shows the steps involved in predicting a target property using ACE-GCN. Each adsorbate surface configuration is initially split into subgraphs (Fig. 1b(1)), which are in turn undirected ‘ego-graphs’ centered around a particular adsorbate generated using the SurfGraph algorithm. These subgraphs explicitly account for the local chemical and structural environment of the adsorbate and can accurately represent the complexities arising from the presence of local coadsorbates, defect sites, and compositional variations. They provide the flexibility to model chemical environments independent of the symmetry, shape, and size of the unit cell, making them useful representations for understanding the influence of local structural features and adsorbate-adsorbate interactions on the binding energy of a given adsorbate. In addition, we note that, for metal systems, it has generally been shown that local arrangement of atoms around the binding site strongly influences the binding energetics. Subgraphs, and the associated connectivity in the graphs, are capable of naturally and implicitly accounting for this effect, while other featurization schemes must explicitly encode this information in the graph structure^6,7,30,50,51^.

After delineation of the subgraphs, every node and edge attribute is expanded as a vector representation of the user-defined chemical and geometric features (Fig. 1b(2)). To systematically capture the geometric and chemical environment features surrounding every node, the node feature vector for each node in a subgraph is iteratively updated based on the neighboring environment through multiple rounds of graph convolution (message-passing) steps (Fig. 1b(3)). Next, hierarchical pooling-like operations are performed to condense multiple arbitrary-sized subgraphs into a fixed-length vector fingerprint (Fig. 1b(4)). This strategy allows ACE-GCN to successfully operate on cases containing arbitrary numbers of adsorbates and associated neighbors. Finally, the fingerprint vector is used as an input to a fully connected neural network to predict the property of interest, such as the average adsorption energy (Fig. 1b(5)). Additional information regarding the attributes considered for chemical and geometric encoding, the graph convolution equation, supplemental indexing, and hierarchical pooling operations is provided in the Methods section.

### Modeling complex heterogeneous catalytic systems using the ACE-GCN scheme

We consider two representative heterogenous catalytic reactions to illustrate the application of ACE-CGN. First, we analyze the stability of high coverage configurations of NO* (‘*’ represents an adsorbed moiety) adsorbed on a Pt_3_Sn(111) surface, and second, we determine the most energetically favorable high coverage configurations of OH* adsorbed on Pt(221) and Pt(100) surfaces. Below, we briefly describe the features of the ACE-CGN algorithm that are highlighted in each example, and in subsequent sections, we provide details of the results.

**Fig. 2:**
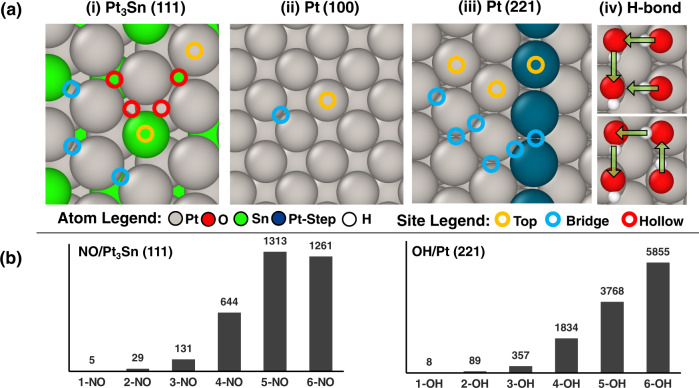
Catalyst configurations analyzed with ACE-CGN. **a** Structural motifs considered in the catalyst models: (i) alloying (Pt_3_Sn(111)), (ii) diversity of binding sites on Pt(100) and (iii) Pt(221) (terrace in gray, step in blue) surfaces, and (iv) directionally dependent intermolecular interactions between adsorbates, such as OH*. Green arrows show the direction of H-bonding for each hydroxyl group. **b** The total number of unique surface configurations, as a function of adsorbate coverage, for Pt_3_Sn(111) and Pt(221). All configurations are generated using the SurfGraph algorithm. Source data are provided as a Source Data file.

The first example demonstrates how the concepts of crystal graph generation and neural network analysis can accelerate the study of the large configurational spaces arising from the presence of high coverages of adsorbates (in this case, NO*) on multi-elemental alloy surfaces. Both surface and bulk alloying introduce a plethora of surface adsorption sites, thereby decreasing the symmetry of the surface and increasing the number of distinct adsorption configurations. As shown in Fig. 2a, for even a single NO* adsorbate, twice as many distinct adsorption configurations exist on Pt_3_Sn(111) as on a pure Pt(111) surface, and this configurational space increases exponentially as the coverage of surface adsorbates increases (Fig. 2b, left). Considering between 1 and 6 NO* molecules, corresponding to surface coverages between 1/12 and 1/2 ML (monolayers), and neglecting sites that incorporate ‘Sn’ atoms, there are approximately 3400 unique adsorbate configurations. A recent publication explored this NO/Pt_3_Sn(111) phase space using an evolutionary algorithm-based scheme, and the present work leverages this prior experience to test and validate the ACE-GCN workflow^17,23^.

The second example demonstrates how high coverage configurations of adsorbates may be enumerated on surfaces with defects, such as steps and non-hexagonal geometries. This case, which focuses on OH*, explicitly considers the effect of adsorbate directionality, stemming from intermolecular hydrogen bonding, on the configurational space. Figure 2a(iii) shows a top view of the Pt(221) step surface, which has a three-atom wide terrace resembling the Pt(111) surface. The number of possible OH* configurations on Pt(221) is significantly larger than that on terrace models such as Pt(100) (Fig. 2a(ii)) or Pt(111), since each row of Pt atoms in Pt(221) has a unique coordination environment, necessitating separate consideration of adsorption sites on each row of Pt atoms parallel to the step edge. Additionally, for given OH* positions on the surface, several hydrogen bonding networks are possible, and since each may have a distinct energy^52^, it is important to explicitly enumerate all such networks (Fig. 2a(iv)). For this purpose, we use directed graphs as an efficient means of incorporating adsorbate directionality and sampling unique H—bond orientations for a given set of OH* adsorbates. Initially, all possible O-O pairs that can form hydrogen bonds are determined, following which all unique hydrogen bonded networks amongst the different pairs are estimated (see Methods section for additional information). Every hydrogen bond, for the adsorbate-centered subgraphs, is explicitly encoded as an additional edge attribute when generating the undirected subgraph for the ACE-GCN. An illustrative example is presented in Fig. 2a(iv), where two possible H-bonding configurations for 4-OH* on Pt(221) are shown. Figure 2b (right), in turn, shows the histogram of the number of configurations as a function of OH* coverage, which were generated by considering both top and bridge sites for configurations with up to three OH* moieties per unit cell (coverage of 1/4 ML), while subsequently, for the cases of 4, 5, and 6 OH* per unit cell (1/3, 5/12 and 1/2 ML, respectively), only top sites are added to the top/bridge configurations of the 1/2/3 OH* structures. The total configurations number approximately 12000, while 1834, 3768, and 5855 configurations are found for the 4, 5, and 6 OH* cases (1/3, 5/12 and 1/2 ML coverages), respectively. As described further below, we use ACE-GCN to efficiently probe these complex configurational spaces, and we additionally illustrate how the approach can be used to combine insights from diverse datasets, in a strategy reminiscent of transfer learning^43–45^, by including OH* adsorption on the geometrically distinct Pt(100) surface, to yield improved predictions.

### Estimating the most relevant high coverage configurations of NO* on a Pt_3_Sn(111) alloy catalyst

**Fig. 3:**
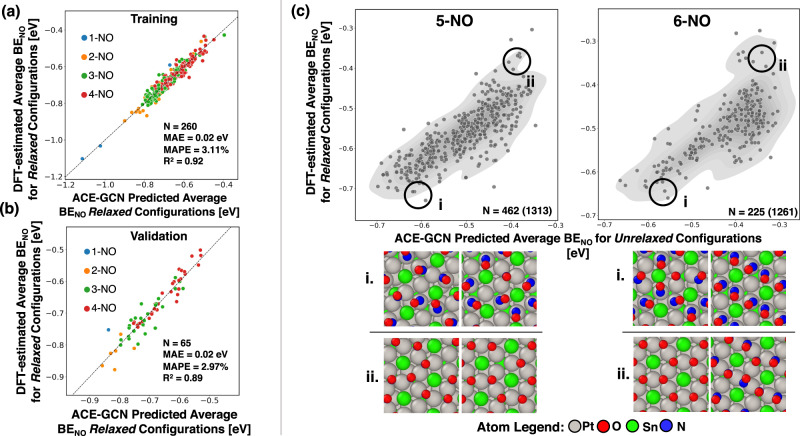
Screening high coverage NO* configurations on Pt_3_Sn(111). Configurational analysis of NO* adsorption on Pt_3_Sn(111), where ACE-GCN is used to predict energetics of the unrelaxed configurations generated using SurfGraph. (**a**) and (**b**) correspond to training and validation parity plots for an ACE-GCN model with NO* configurations consisting of 1–4 NO* molecules per unit cell. Test set performance and training related to configurations with 4 NO* molecules per unit cell are discussed in the Supplementary Information, Fig. S8. (**c**) gives predictions of the ACE-GCN model, trained on configurations of 1–4 NO* molecules per unit cell, for stability of *unrelaxed* 5 and 6 NO* configurations generated with SurfGraph. The predicted average BE of the unrelaxed configurations is plotted on the *x*-axis, while the final energy of the same configurations after DFT relaxation is plotted on the y-axis. Only configurations where the binding location of the NO* did not change after DFT relaxation are included. The ACE-GCN algorithm successfully predicts trends in adsorption energies based solely on the unrelaxed configurations generated by SurfGraph. A representative area of chemical space relevant for unstable and stable configurations is depicted on the scatter plot (**c**). Selected relaxed low- and high-energy configurations are shown in insets (i) and (ii), respectively. Source data are provided as a Source Data file.

As shown in Fig. 2b(i), the total number of unique initial configurations for 1–6 NO* molecules adsorbed on a $\sqrt{12}\times \sqrt{12}$ Pt_3_Sn(111) unit cell (coverage range of 1/12–1/2 ML) is on the order of ~3400, with roughly 2500 configurations for the 5 and 6 NO* cases (5/12 and 1/2 ML coverages) alone. The goal of the proposed screening strategy is to develop a surrogate model that describes the key interactions governing the stability of low coverage NO* configurations (1/2/3/4 NO* per unit cell) and to use the resulting insights to efficiently screen the vast number of higher coverage configurations (5/6 NO* per unit cell) with minimal additional computational effort. First, an ACE-GCN model is trained on the average NO* binding energies of all the low coverage (1, 2, and 3 NO*, or 1/12 to 1/4 ML), DFT-relaxed structures (details of the 1–3 NO* model fit are provided in Supplementary Information S4), and next, the model is used to predict binding energetics for the 4 NO* (1/3 ML) case. Based on these ACE-GCN predictions, 100 energetically stable and 100 unstable candidates (200 in total) of the 644 possible 4 NO* configurations are then selected. These configurations are relaxed using DFT and added to the incremental model training. Figure 3a, b shows the parity plots for the training and validation sets for the new 1/2/3/4 NO* dataset. The model fits the target property, the average NO* binding energy, with a mean absolute error of 0.02 eV for training and validation sets and a mean absolute error of 0.02 eV on the test set comprised of 4 NO* data points not used in the training (Fig. S8), demonstrating that the ACE-GCN architecture can distinguish amongst different coverages through representations consisting of subgraph-based graph convolutions and hierarchical pooling. More information on the performance is shown in Fig. S8 of the Supplementary Information. Next, the modified ACE-GCN model, trained on the exhaustive 1/2/3 NO* ensemble and some 4 NO* data points, is used to rank the unrelaxed 5 NO* and 6 NO* configurations (5/12 and 1/2 ML coverages), generated through SurfGraph, as shown in Fig. 3c. This dataset is comprised of 1314 and 1261 configurations for 5 and 6 NO*, respectively. In the figure, the x-axis represents the ACE-GCN predicted average binding energy of the initial, unrelaxed 5/6 NO* configurations, and the y-axis gives the corresponding DFT-relaxed energy. For comparison, we also analyze the performance of ACE-GCN on predicting the average binding energies of the relaxed 5/6 NO* configurations when only the low coverage 1, 2, and 3 NO* cases are used for training (see Supplementary Information Figs. S9, S10). We find that at least some 4 NO* points are important for predicting the energies with reasonable accuracy, while the proportion of 4 NO* points included in the training also has a modest impact on the final model performance. We note that, for clarity, only those NO* configurations whose binding locations did not change after DFT relaxation are plotted; additional discussion is provided in Supplementary Information S4.

Importantly, the top 10% lowest energy unrelaxed configurations identified by ACE-GCN include the most stable DFT-relaxed atomistic configurations for both the 5 and 6 NO* cases, and no additional stable configurations were found after DFT relaxation that were not already identified by SurfGraph (see Supplementary Information for additional details). These results, taken together, strongly suggest that the combination of SurfGraph and ACE-GCN is capable of efficiently identifying all stable high coverage configurations for NO* adsorption.

The ACE-GCN model also captures important information regarding the governing interactions dictating the adsorption geometries of NO* on Pt_3_Sn(111). From our recent analysis^23^, it is known that higher coverages of NO* are stable in mixed top and bridge configurations on this surface, while combinations of bridge and threefold sites are unstable. The ACE-GCN model captures this insight, without any explicit user input, using only the low coverage (1/2/3/4 NO*, 1/12 to 1/3 ML) data, and, as described above, efficiently identifies the energetically most stable 5 NO* and 6 NO* (5/12 and 1/2 ML) configurations. The low energy configurations, identified as the most negative adsorption energy configurations in the vicinity of region (i) in Fig. 3c, consist of NO occupying the top and bridge sites on Pt_3_Sn. In contrast, higher energy configurations, identified as the most positive adsorption energy configurations in the vicinity of region (ii) in Fig. 3c, consist of NO* occupying a mixture of bridge and hollow sites, and are also accurately identified by the ACE-GCN surrogate model.

Finally, it is interesting to note that the degree of restructuring of the adsorbate site after DFT relaxation is directly correlated with the stability of a given configuration as predicted using ACE-GCN. The sites predicted to be the most unstable by ACE-GCN underwent the largest change in adsorbate positions after relaxation, and vice versa, suggesting that ACE-GCN predictions of stable structures are likely to remain stable, and largely unreconstructed, after DFT relaxation. Additional discussions of these reconstructed configurations, as well as details of the effect of different training sets on the model’s predictive capabilities, are included in Supplementary Information S4.

These results strongly suggest that, through selective incorporation of a small subset of data points of increasingly higher coverages, the ACE-GCN model, trained primarily on low coverage configurations (1–4 NO*, 1/12–1/3 ML), successfully identifies stable high coverage configurations (5/12–1/2 ML) based solely on the unrelaxed geometries generated from SurfGraph. In comparison to the evolutionary algorithm (EA) scheme used in our previous work, the ACE-GCN model (i) required fewer DFT calculations (350 versus over 500 data points) than the EA^23^, and (ii) independently identifies key chemical and geometric information affecting the adsorption energetics. This is an important advantage that becomes even more significant for larger chemical spaces, where careful analysis of individual configurations and development of chemical intuition becomes less practical.

### Identifying stable high coverage configurations of interacting hydroxyl adsorbates on defected Pt surfaces

This case study illustrates the application of our proposed workflow to adsorbates with directionally dependent hydrogen bonding on the non-hexagonally close-packed single crystal surfaces, Pt(100) and Pt(221). The former is chosen as the simplest possible non-hexagonal surface, while the latter represents model step defects that have been shown to exert a significant influence on electrochemical oxygen reduction rates on polycrystalline Pt electrocatalysts^40,53^. In addition to discussing a comprehensive training/testing/extrapolation strategy for the Pt(100) and Pt(221) surfaces, similar to that discussed for the NO/Pt_3_Sn(111) case study, we additionally explore the ability of the ACE-GCN framework to synergistically combine insights from training datasets from these two surface morphologies (the benefit of considering such a mixed training dataset is further discussed in the Supplementary Information S5). Such strategies will ultimately be key to understanding adsorption configurations on highly complex catalysts, such as polycrystalline nanoparticles, which encompass a variety of different catalyst morphologies^54,55^.

The overall workflow is summarized here and described in more detail in subsequent paragraphs. First, a comprehensive training dataset, consisting of configurations with between 1 and 5 OH* molecules per 8 Pt atoms on the Pt(100) surface (coverages of between 1/8 and 5/8 ML), is generated, while a second training set of between 1 and 3 OH* adsorbed per 12 Pt atoms on Pt(221) (coverages of 1/12 to 1/4 ML) is also created. Although the coverages considered on the stepped surface are much lower than those analyzed on Pt(100), the total number of training data points is very similar in each case. These datasets, through ACE-GCN, are then combined to efficiently identify low energy adsorption configurations of OH* on Pt(221) at much higher coverages (4–6 OH*/12 Pt, coverages of 1/3–1/2 ML), where the total number of configurations is exponentially larger (Fig. 2c) than the number of configurations associated with similar coverages on Pt(100).

**Fig. 4:**
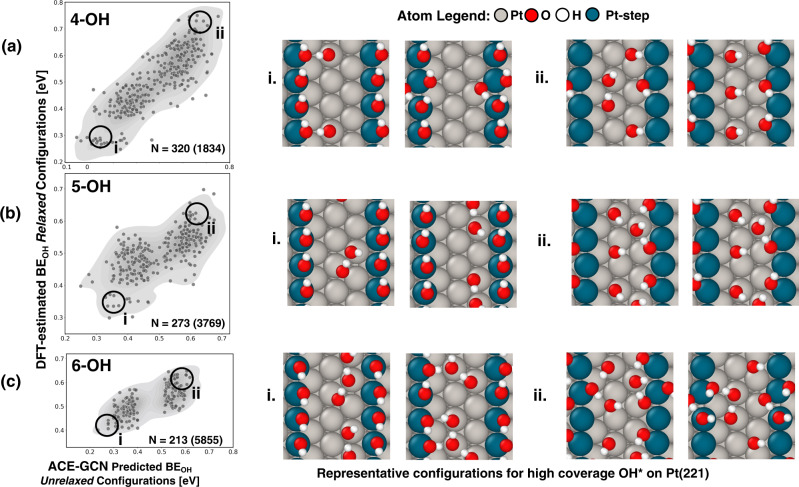
Screening high coverage OH* configurations on Pt(221). Subfigures (**a**), (**b**), and (**c**) show scatter plots for average OH* binding energies of unrelaxed configurations, as predicted by ACE-GCN (x-axis), with respect to DFT-relaxed energies of the corresponding structures (y-axis). A representative area of chemical space relevant for unstable and stable configurations is depicted on the scatter plots (marked ‘i’ and ‘ii’). Numbers in the inset show the total number of DFT-relaxed configurations compared to the total possible structures enumerated by SurfGraph. The ACE-GCN model for each succeeding coverage (4/5/6 OH* per unit cell, 1/3 to 1/2 ML) is trained on configurations with lower coverages (see text for details). On the right side of the scatter plots, representative stable and unstable atomic configurations, from regions ‘i’ and ‘ii’ depicted in the scatter plots, are shown. A few relaxed configurations showing OH* species dissociation after DFT relaxation were not included in the plots or model retraining (analysis of dissociated configurations is discussed in the Supplementary Information S5). Source data are provided as a Source Data file.

The OH* configurations are generated using a modified SurfGraph code that accounts for directional hydrogen bonds among different OH* species (see Fig. 2a for an example). As mentioned above, the ACE-GCN model is initially trained on the dataset comprised of configurations between 1–3 OH* adsorbates per unit cell on Pt(221) and 1–5 OH* per unit cell on Pt(100). Next, ACE-GCN is used to rank the unrelaxed 4OH* (1/3 ML coverage)/ Pt(221) configurations (1834 in total), from which 400 configurations, representing a range of energy values and adsorbate binding configurations, are chosen for full DFT relaxation. Figure 4a (left hand side plot) shows a comparison of the ACE-GCN predicted average binding energies of the unrelaxed 4 OH* configurations and the corresponding DFT-relaxed energies. There is a robust correlation between these two quantities, demonstrating that configurations predicted to be low (or high) in energy based on the ACE-GCN predictions of initial unrelaxed geometries track well with post-DFT relaxation results. Shown on the right of the scatter plot are selected 4 OH* configurations (marked as ‘i’ and ‘ii’), after DFT relaxation, belonging to the low/high energy 4 OH* arrangements. The most stable structures, identified as the most negative adsorption energy configurations in the vicinity of region (i) in the plot, have the Pt step edge (marked in dark blue) completely occupied, with any additional OH* moieties have clustered around the Pt edge to increase the level of hydrogen bonding. In contrast, the high energy structures, identified as the most positive adsorption energy configurations in the vicinity of region (ii) in the plot, are comprised of separated OH* species, most of which are not directly adsorbed on the Pt step edge, and with relatively few hydrogen bonds. These results indicate that the ACE-GCN model, trained on the diverse data from Pt(100) and Pt(221), accurately learns the underlying features that stabilize the 4 OH* configurations on Pt steps.

Following the scheme laid out in Fig. 1a, higher coverage (5 OH*, 5/12 ML coverage) configurations are generated by using SurfGraph to systematically add an additional OH* moiety to the exhaustive set of unrelaxed -OH* configurations. These configurations are then ranked using a retrained ACE-GCN model incorporating the previously DFT-relaxed 4 OH* configurations in the training set. A few of the identified configurations resulted in dissociated OH* species after relaxation, and these cases have not been included in the analysis or model retraining (see Supplementary Information S5). Analogous to the 4 OH* case, a total of 400 unrelaxed configurations, 200 each chosen from high and low energy zones as identified by the ACE-GCN predictions, are selected for DFT relaxation. Finally, a similar strategy is applied when searching for 6 OH* configurations (1/2 ML of coverage), where the emphasis is again placed on high- and low-energy structures. 3769 and 5855 possible OH* configurations exist for the 5 and 6 OH* cases, respectively, of which only about 400 configurations each for 5 and 6 OH* cases are evaluated using DFT, and about 273 and 213 cases remain undissociated after DFT relaxation. The correlation between the stability of structures predicted via ACE-GCN and those after DFT optimization is again quite reasonable (left side scatter plots in Fig. 4b, c). The quasi-bimodal nature of the 5 and 6 OH* plots is simply the result of our choice to sample high and low energy structures, as predicted by ACE-GCN, for DFT optimization. Further, in line with the chemical intuition developed with lower coverages, as shown in insets ‘i’ and ‘ii’ on the right side of Fig. 4b, c, for both 5 and 6 OH* cases (5/12 and 1/2 ML coverages), the most stable configurations are comprised of clustered OH* species on the Pt-step edge, whereas unstable cases involve spatially separated OH* with few OH* moieties adsorbed on the step edge. We note, however, that despite the reasonable energetic and chemically intuitive predictions from the ACE-GCN analysis, there can be non-trivial changes during relaxation of the unrelaxed structures, especially for the high coverage cases with 5 and 6 OH* adsorbates per unit cell (5/12 and 1/2 ML). We attribute these relaxations to the observation that multiple highly clustered OH* representations may have similar average OH* interaction energies but may, nevertheless, undergo substantially different relaxation during DFT optimization. In the future, we expect that classification algorithms, with the ability to predict the initial configurations that could most likely undergo restructuring or dissociation, could be developed to more efficiently characterize the full configurational space.

The Pt(221) and Pt(100) analyses demonstrate the capability of ACE-GCN to (i) learn important underlying interactions governing the stability of adsorbates with directionally dependent interactions, such as OH*, on irregular catalyst models by simulating only about 5–6% of the total number of possible configurations, and (ii) combine data having different catalytic morphologies, in a transfer learning-inspired approach, to train surrogate models with high efficiency. Such an analysis can aid in developing chemical intuition regarding the underlying interactions that are crucial for stabilizing the adsorbates and understanding the state of the system in realistic reaction environments. Although not attempted in the present study, we expect that the method could be extended to leverage learning on the Pt(221) surface to more efficiently predict OH* adsorption configurations on Pt-based alloys, as well^56^.

### Mechanistic implications of high OH* coverages for electrochemical reactions on Pt

Based on the identified OH* configurations on the irregular Pt surfaces, a detailed thermodynamic analysis to investigate the state of the catalyst surface under electrochemical reaction conditions, such as those relevant to the oxygen reduction reaction (ORR), can now be undertaken. Previous reports have demonstrated that (111) terraces on Pt catalysts are among the most active facets for ORR, and recent investigations on irregular crystal facets of Pt, having variable step sizes ((221), (331) and (211)), suggest high ORR activity on these surfaces, as well^40,53,57^. A mechanistic analysis incorporating the effects of catalyst morphology and OH* coverages is, in turn, needed to understand these experimentally observed trends. However, the large phase space of possible atomic configurations, especially for the case of stepped catalyst surfaces, makes the analysis highly non-trivial.

**Fig. 5:**
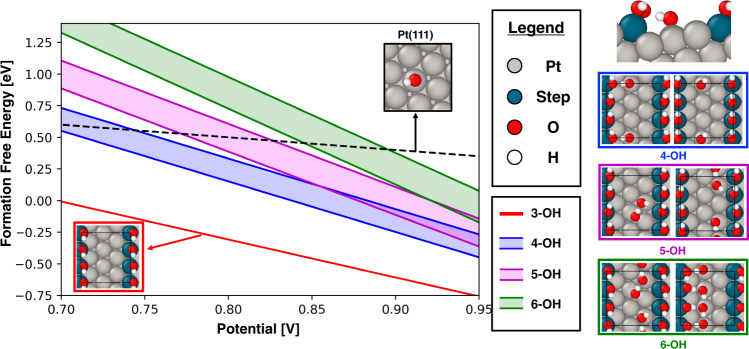
Ab initio Pourbaix diagram based on binding energies of various OH* configurations on Pt(111) and Pt(221) surfaces. In the potential region of interest for ORR, 0.8–0.9 V, competition between ensembles having 4, 5, and 6 OH* adsorbates per unit cell on Pt(221) (1/4 to 1/2 ML OH* coverage) is predicted and is shown by the overlapping free energy bands. On the rightmost side of the figure, a side view of the most stable 4 OH* configuration in our analysis is shown (the corresponding top view is in the 4 OH inset on the left side). It is observed that the OH* present on the terrace site immediately below the Pt step edge (termed “back terrace” in our discussion) has a favorable hydrogen bond with the OH* absorbed on the edge. Such an arrangement of OH* moieties is possible due to the particular geometry of the step and edge sites. This arrangement results in appreciable stabilization compared to the scenario where no such hydrogen bonding exists (shown in the 4 OH* inset, right side). Representative surface configurations for 5 and 6 OH* are also indicated on the insets to the right of the figure, with the most stable configurations on the left of the insets. Source data are provided as a Source Data file.

Utilizing the results generated in the previous section, an ab initio surface Pourbaix diagram is generated (Fig. 5) to explain the state of the Pt(221) surface under ORR-relevant conditions. For simulations reported in Fig. 5, larger unit cells, along with higher energy cutoffs and k-points, are utilized, with additional details reported in the Methods section. The formation free energies of the identified high coverage structures (4–6 OH* on Pt(221)) are plotted as a function of the applied external potential vs. the Standard Hydrogen Electrode (SHE). The formation free energy for each OH* coverage is presented as an energy band, which is approximately 0.25 eV wide, starting from the energy of the most stable configuration identified using the workflow shown in Fig. 1. The schematics on the right side of the Pourbaix diagram show the most stable and selected metastable (approximately 0.25 eV higher in energy) configurations. It is observed that the relative shift in energetics between the most stable and the corresponding metastable structures is on the order of 0.05 eV when comparing the simulations performed on smaller cells, with fewer k-points, and a lower plane-wave energy cutoff, with those performed on larger cells, higher k-point density, and higher planewave energy cutoff. In addition, the free energy of the most stable 3 OH* configuration on the Pt(221) facet, together with that of a single OH* moiety on Pt(111), is plotted for reference. The 3 OH* ensemble on Pt(221), where the OH* species occupy the Pt step edge, is identified as the most stable OH* configuration. This result suggests that the Pt edge might be completely poisoned under ORR-relevant conditions (red inset and line in Fig. 5). Additional population of OH* on the surface of the catalyst (4, 5, and 6 OH*) shows competition amongst different configurations, especially above applied potentials of 0.8 V vs. SHE. An interesting feature of the identified high coverage configurations on Pt(221) is the presence of the OH* adsorbed on the terrace sites that lie adjacent to, and below, the Pt step edge. Such a binding configuration is a result of the unique spatial arrangement of Pt(221) step sites (a representative configuration is shown in Fig. 5, right side, top inset). Discovering such a unique OH* binding arrangement, which, to the best of our knowledge, has not been reported elsewhere, speaks to the value that data-driven screening workflows such as ACE-GCN can add in helping to identify interesting regions in the chemical phase space that can then be further explored to better understand the complex reaction systems.

It is interesting to observe that multiple possible H-bonding arrangements can possess comparable energies. The most stable OH* arrangements often exhibit hydrogen bonding between the OH* moiety on the lower terrace and the OH* adsorbed on the Pt edge (Fig. 5, inset for 4 OH* case), or they possess a combination of OH* adsorbed on both bridge and top sites in chain-like structures near the step on the upper terrace (Fig. 5, inset for 5 and 6 OH*).

It is important to note that, while the identified structural motifs for high coverage adsorbed OH* may be relevant to practical ORR catalysis, these configurations only consider stabilization due to adsorbate-adsorbate and adsorbate-substrate interactions and do not explicitly account for interactions between adsorbed hydroxyl groups and ambient water solvent molecules, which can have energies on the order of 0.5–0.6 eV per OH*^52,58,59^. To illustrate the effect of such corrections, a black dashed line, representing the OH* adsorption energy on Pt(111), is plotted in Fig. 5. At an applied potential of 0.8 V vs SHE, the formation free energy for 1 OH* adsorbed on a top site of Pt(111) is 0.55 eV, excluding any solvent corrections, which is consistent with previous reports. It is only the solvent stabilization that reduces the energy of OH* to near zero on Pt(111) (at 0.8 V vs. SHE) and hence promotes its reactivity. Since the energy of 1-OH* on Pt(111), devoid of any solvent correction, is comparable to the uncorrected energy of the 4/5/6 OH* ensembles on Pt(221), one might expect that some of these ensembles on Pt(221) would be stabilized under ORR condition and contribute to the ORR activity. Further, it is possible that the solvation correction for the high coverage 4/5/6 OH* cases (1/4 to 1/2 ML of OH*) on Pt(221) could be different compared to the correction for the low coverage OH* ensembles on Pt(111). To fully capture the impact of solvent-adsorbate interactions on ORR chemistry, further analysis, rigorously incorporating explicit solvent molecules (H_2_O), along with ab initio molecular dynamics analysis to understand the electrode-electrolyte double layer structure, would be necessary (it might, indeed, be possible to combine the ACE-GCN framework with machine learning-based force fields to understand such dynamical effects)^60–62^. The identified 4/5/6 OH* high coverage configurations provide a strong foundation for undertaking such an analysis, and it is not difficult, in principle, to add water molecules as coadsorbates amongst the OH* moieties. Nevertheless, given that OH-OH and OH-H_2_O interactions are of comparable magnitude, many of the key qualitative conclusions from the analysis, such as the favorable adsorption of OH* on the step edges and the preference for OH* on the lower terrace to interact with the step-adsorbed OH* groups, are unlikely to be altered by the presence of additional water molecules^52^.

In conclusion, we present a machine learning-based hierarchical screening workflow to systematically estimate surface adsorption structures for complex heterogeneous surface catalytic reactions. The proposed workflow utilizes the graph theory-based SurfGraph algorithm for systematic enumeration and generation of surface adsorbate representations with variable coverages. The generated models are screened using the Adsorbate Chemical Environment-based Graph Convolution Neural Network (ACE-GCN), a graph neural network-based framework, which utilizes the chemical and structural environment of a given adsorbate as the input and maps these features to the target property of choice. Using this workflow, we demonstrate the identification of relevant surface models for heterogeneous catalytic systems comprised of strong binding adsorbates on low symmetry alloyed surfaces and for directionally dependent adsorption on defect surface structures. In both cases, our model successfully identifies trends in the relative stability of different atomic configurations at a fraction of the computational cost (~10%) of exhaustive DFT calculations, thereby providing a framework to identify relevant atomic configurations for surface environments with large and complex configurational spaces. In addition to reducing the overall computational cost, this automated approach reduces the possibility of systematic bias resulting from the use of chemical intuition alone to identify structures with target properties. This approach can therefore serve as a starting point for developing a detailed atomic description of complex catalyst surfaces under in-situ conditions, and help identify interesting regions of the chemical solution space to be investigated with rigorous state-of-the-art methods, ultimately leading to fundamental insights into factors that govern heterogeneous catalysis in structurally and chemically complex environments. In the future, such an approach, combined with configuration sampling schemes deploying uncertainty-driven predictions and workflows inspired from active-learning strategies, would aid in even more efficient selection of relevant atomistic configurations^63,64^.

## Methods

### Dataset

The dataset used for model training and prediction is a collection of a diverse set of calculations corresponding to 1) NO*, varying from 1–6 adsorbates (coverages of 1/12 to 1/2 ML), on a Pt_3_Sn(111) surface, and 2) OH* surface configurations on Pt(100) and Pt(221), also encompassing 1–6 adsorbates per unit cell (coverages of 1/12 to 1/2 ML)—see below for unit cell details). The graph enumeration code, SurfGraph^23^, is used to identify the binding sites and to generate the high coverage configurations which are converted to a graph object through ACE-GCN for property prediction. The target property of choice is the binding energy of the adsorbates, normalized to the number of adsorbates considered in the facet:

1 $${{{{{\rm{B}}}}}}{{{{{{\rm{E}}}}}}}_{{{{{{{\rm{NO}}}}}}}}=\frac{{E}_{n{\mbox{-}}{{{{{{\rm{NO}}}}}}}/{{{{{{\rm{Slab}}}}}}}}-{E}_{{{{{{{\rm{Slab}}}}}}}}-{{nE}}_{{{{{{{\rm{NO}}}}}}}\left({{{{{\rm{g}}}}}}\right)}}{{n}_{{{{{{{\rm{NO}}}}}}}}}$$

2 $${{{{{\rm{B}}}}}}{{{{{{\rm{E}}}}}}}_{{{{{{{\rm{OH}}}}}}}}=\,\frac{{E}_{n{\mbox{-}}{{{{{{\rm{OH}}}}}}}/{{{{{{\rm{Slab}}}}}}}}+\frac{n}{2}{E}_{{{{{{{\rm{H}}}}}}}_{2}\left({{{{{\rm{g}}}}}}\right)}-{E}_{{{{{{{\rm{Slab}}}}}}}}-n{E}_{{{{{{{\rm{H}}}}}}}_{2}{{{{{\rm{O}}}}}}\left({{{{{\rm{g}}}}}}\right)}}{{n}_{{{{{{{\rm{OH}}}}}}}}}$$

### DFT methods

The simulations for NO* on Pt_3_Sn(111) were adopted from the previous publications^23^. For the case of OH* adsorption on Pt(221), the simulations are performed within the framework of periodic density functional theory with the Vienna Ab Initio Simulation Package (VASP)^65^. The energies and geometries of the most stable configurations of OH* on the Pt(221) surface are obtained through minimization of the total energy with respect to geometry by spin-polarized generalized gradient approximation calculations (GGA-PBE)^66^. The projected augmented wave (PAW) method is used to account for the effect of core electrons on the valence electron density^67^. A PBE-calculated lattice constant of 3.97 Å for pure Pt is employed. The Pt(221) surface is represented by a 3 × 3 unit cell with 4 layers (total of 33 atoms per unit cell). A vacuum equivalent to 13 Å is applied between any two successive slabs, and surface relaxation is allowed in the top three layers. A planewave energy cutoff of 300 eV is used for the high-throughput calculations. A minimum k-point grid sampling of 3 × 3 × 1 is employed. For selected cases reported in the phase diagram in Fig. 5, a larger unit cell containing 60 Pt atoms is utilized, and a planewave energy cutoff of 400 eV, along with k-point grid sampling 4 × 4 × 1, is employed. It is observed that between the two different kinds of models and simulation parameters utilized, the trends in the adsorption energies of OH* remains the same, with minimal (~0.1 eV) change in differential adsorption energies between any two subsequent coverages, and a change in the order of 0.05 eV between relative adsorption energies of different configurations for a given coverage. The electronic occupancies are determined according to a Methfessel− Paxton scheme with an energy smearing of 0.2 eV. Dipole corrections are used in all cases to decouple the electrostatic interactions between the periodically repeated slabs. Structures are fully relaxed until the Hellmann− Feynman forces acting on the atoms are smaller than 0.05 eV/Å. Atomic configrations are visualized using Atomic Simulation Environment (ASE) and Ovito^68,69^.

### Adsorbate subgraph generation

Adsorbate subgraphs are generated using the SurfGraph algorithm^23^. Initially, for a given unit cell, a full graph incorporating all the atoms in the cell is generated. Adsorbate nodes are then identified, and a subgraph is generated with each identified adsorbate node as the center. The subgraphs are generated such that they incorporate the information of the surface atoms immediately adjacent to the adsorbate along with other adsorbate atoms interacting with these surface atoms.

### Hydrogen bond generation with directed graphs

All hydrogen atoms with a bond distance greater than 1.3 Å and less than 2.1 Å to a given oxygen atom are classified as hydrogen bonds. To construct combinations of possible pairs of hydrogen bonds between a set of oxygen atoms, all such possible bonds are identified using the rule explained in the previous sentence. Then, all possible directed graphs are generated between the identified pairs, using the rule that each OH adsorbate can only donate one hydrogen bond and accept multiple hydrogen bonds. The directed graph combinations with the maximum number of hydrogen bond pairs are then selected for property prediction or to perform DFT simulations.

### Model architecture and implementation

Graph neural networks (GNN), also known as message-passing neural networks^70^, have been previously proposed for computer vision, natural language processing, generating molecular fingerprints, predicting crystal bulk properties, and predicting binding energy on surface slab models. The network developed in this work is the extension of the graph convolution neural network (GCN) approach introduced by Xie et al^32^. The GCN framework is coupled with a subgraph generation routine to systematically encode complex high coverage surface configurations. The subgraphs capture important features of the high coverage geometries, and at the same time, the versatility of the neural networks provides nonlinear mapping between the chemical fingerprints and the target property. In this work, the subgraphs are selected to encode interactions out to the second nearest neighbor for a given adsorbate, including the interactions with the active site (1st neighbor) and with the catalyst and adsorbate atoms surrounding the active site (2nd nearest neighbor). These boundaries are chosen since only short-range interactions are important for the adsorbates considered in these cases, but we note that the representations are sufficiently flexible to account for other long-range interactions, as needed. A few, initial, DFT simulations may be required to calibrate the spatial extent of adsorbate-adsorbate interactions and hence determine the size of subgraphs needed for initial model training, but the number of such calculations is generally very small compared to the computational savings derived from the overall machine learning framework. With these approaches, it is possible to strike a balance between end-to-end feature learning, provided by deep neural networks, and chemical intuition found in ‘hand-engineered’ features.

Each crystal lattice entry is split into smaller network motifs (subgraphs) as per the number of unique adsorbates identified by SurfGraph^23^. Each subgraph is an adsorbate-centered undirected graph (ego-graph) with nodes representing the atoms and edges representing the connection between the neighboring atoms in the lattice. The chemical identity of each node in this subgraph is represented by a feature vector generated based on its elemental identity using a combination of chemical and geometric features. These attributes are encoded as one-hot encoding. The edge connecting two nodes is described by edge attributes based on the spatial pairwise atom distance. This feature can be expressed either as a Gaussian feature expansion, as proposed in the original implementation^32^, or as one-hot encoding, as implemented in the current version. The reason for using the one-hot encoding expression of the spatial bond distance is to modulate model’s sensitivity to bond fluctuations arising out of structure optimization. A full list of chemical and geometric properties used is provided in Supplementary Information S2. Next, the bond distance and the one-hot encoding are used to create an adjacency matrix for each subgraph. An indexing scheme is generated to account for various neighbors of a given node; each node index is superseded by the adsorbate index based on the number of unique adsorbates in each crystal entry. Likewise, for every node atom and its corresponding neighbors, the atom indices are superseded by supplemental indexing linking the neighboring atoms to its parent node. This indexing strategy facilitates the subsequent hierarchical pooling operations, enabling the network to account for arbitrary-sized subgraphs. A schematic of this pooling operation strategy is provided in Supplementary Information S2. Model training starts by embedding node attributes in subgraph embeddings. The graph convolution layers iteratively update the node feature vectors by performing convolutions with surrounding nodes in the subgraphs using

3 $${Z}_{{\left(i,j\right)}_{k}}^{\left(t\right)}={v}_{j}^{\left(t\right)}\oplus {u}_{{\left(i,j\right)}_{k}}$$

4 $${v}_{i}^{(t+1)}={g}_{{{{{{\rm{act}}}}}}}\left[\left(\mathop{\sum }\limits_{j,k}^{N({v}_{i}),\,E({v}_{i})}{W}_{c}^{(t)}{Z}_{{(i,j)}_{k}}^{t}\right)+{W}_{s}^{(t)}{v}_{i}^{(t)}+{b}^{(t)}\right]$$

5 $${g}_{{{{{{{\rm{act}}}}}}}}\left(x\right)={{{{{{\rm{ln}}}}}}}\,{{{{{{\rm{ln}}}}}}}\left(1+{e}^{x}\right)$$

6 $${V}_{G}=\frac{1}{{N}_{P}}\mathop{\sum }\limits_{P}^{{N}_{P}}{V}_{G}^{(P)}$$

Equation (3) is the new fingerprint vector formed by concatenation of corresponding neighbor and edge features for each node. Equation (4) shows the graph convolution equation used for iterating the node features in each message-passing round. This equation is inspired from work for predicting small molecule and bulk crystal properties^32^. Here, ${W}_{x}$ and $b$ are the shared weights and biases for the graph convolution module, while *g*_act_ (Eq. 5) is the softplus activation function, a smooth approximation of the ReLU (rectified linear unit). Equation (6) shows the read-out phase of the learning, wherein the node embeddings for different subgraphs for every adsorbate are combined into a single vector.

The hierarchical pooling is implemented using PyTorch- scatter module’s scatter method^71^. Through this method, elements in the input matrix of known dimensions can be reduced (summed or normalized) by explicitly specifying the indices which have been used for said reduction. As a result, arbitrarily sized subgraphs are collapsed into a single user-defined n-sized vector fingerprint equivalent to the atom embeddings defined for each atom node at the start. Following the convolution and mean pooling operations, the fingerprint vector is supplied to fully connected layers to capture the mapping of configuration to the target property. The creation of graph objects for the high coverage configurations is parallelized across multiple CPU cores using DASK^72^.

### Model training

The network performance is evaluated using three common metrics based on the model’s residuals, the mean absolute error (MAE), the root mean-squared error (RMSE), and the mean absolute percentage error (MAPE). A (80/10/10) train-validation-test scheme is adopted for choosing the best model for prediction. During the training phase, the data is randomly split into a train-validation-test split where the test set is kept aside for final evaluation. Data drift and split stochasticity were analyzed by considering different random seeds in the data splitting, but there was no discernable impact on the model performance. The model weights are iteratively updated by minimizing the loss function (MSE in this case) associated with predicting the target in the training data, and the validation set is scored after each epoch (as per the MAE). The Adam optimizer as implemented in PyTorch is used for the training. After model training for predefined epochs, the model with the best validation score is selected for evaluation on the test set. In-house implementation of a grid search method was used to find the best set of hyperparameters for each ACE-GCN instance. The initial estimates for the parameters were adopted from Xie et al.^32^. A complete list of hyperparameters is provided in Supplementary Information S3. Model training and validation were carried on local CPU cores and Tesla P100 GPU cores provided by Purdue’s Research Computing Facility.

## Supplementary information

Supplementary Information

## Data Availability

The optimized high coverage configurations in form of raw file and process datasets for NO-Pt3Sn(111), OH-Pt(100) and OH-Pt(211) are available at: Pushkar Ghanekar, Siddharth Deshpande, Jeffrey Greeley, *Adsorbate chemical environment-based machine learning framework for heterogeneous catalysis*, Materials Cloud Archive 2022.50 (2022), 10.24435/materialscloud:td-hf.

## Code availability

The ACE-GCN code as implemented using PyTorch 1.7^73^ is available from: https://gitlab.com/jgreeley-group/ace_gcn made available under MIT license.

Ghanekar, Pushkar, Deshpande, Siddharth, & Greeley, Jeffrey (2022). Adsorbate chemical environment-based machine learning framework for heterogeneous catalysis. Zenodo. 10.5281/zenodo.7023990
