# Supplementary material for: Adsorbate chemical environment-based machine learning framework for heterogeneous catalysis
Source: Nat Commun. 2022 Oct 2;13:5788. doi: 10.1038/s41467-022-33256-2 (PMC9527237; doi:10.1038/s41467-022-33256-2)
Supplement: Supplementary file 1 — Supplementary Information [file 41467_2022_33256_MOESM1_ESM.pdf]

Adsorbate chemical environment-based machine learning framework for heterogeneous catalysis

Pushkar G. Ghanekar[1*], Siddharth Deshpande[1,2*‡], and Jeffrey Greeley[1‡]

[1] Davidson School of Chemical Engineering, Purdue University, West Lafayette, Indiana 47907, USA

[2] Current address: Department of Chemical Engineering, University of Delaware, Newark, DE

‡ sdeshpan@udel.edu, ‡ jgreeley@purdue.edu

* Equal contribution

‡ Corresponding authors

Supplementary Information

1. Atomic configurations and datasets

For NO on $Pt_3Sn(111)$, we have considered between 1-6 NO* absorbed on a $\sqrt{12} \times \sqrt{12}$ $Pt_3Sn(111)$ unit cell, with a total of 3383 geometric configurations, of which 1994 were identified as unique geometries after DFT relaxation. For the OH/Pt adsorbates, Pt(100), with a 3x3 unit cell, has 169 unique, non-dissociated configurations after DFT relaxation, comprising coverages from 1-OH to 5-OH, while for Pt(221), a 3x3 unit cell was selected, and we have analyzed 454 unique, non-dissociated configurations, with coverages between 1-OH and 3-OH, after DFT relaxation. With 4-OH adsorbates, the graph enumeration scheme identified 1834 configurations, out of which 400 cases (corresponding to the most and least stable configurations) were simulated, from which 320 unique non-dissociated configurations were identified after DFT relaxation. In the case of 5-OH adsorbates, the graph code enumeration scheme identified 3769 structures, out of which 400 cases (most and least stable configurations) were relaxed with DFT, resulting in 273 non-dissociated converged configurations. For 6-OH adsorbates, a total 5855 configurations were identified. From these, 400 configurations (most and least stable) were chosen for DFT evaluation, finally resulting in 213 unique, non-dissociated configurations.

NO/Pt_3Sn (111)		
Type of coverage	# of configurations	
	Configurations from graph enumeration	Unique configurations post-DFT relaxation
1-NO	5	5
2-NO	29	29
3-NO	131	131
4-NO	644	366
5-NO	1313	711
6-NO	1261	752
Total	3383	1994

OH/Pt (221)		
Type of coverage	# of configurations	
	Configurations from graph enumeration	Unique configurations post-DFT relaxation
1-OH	--	8
2-OH	--	89
3-OH	--	357
4-OH	1834[b]	320[a]
5-OH	3768[b]	273[a]
6-OH	5855[b]	213[a]
Total	11457	1260

OH/Pt (100)	
Type of coverage	Unique configurations post-DFT relaxation
1-OH	3
2-OH	8
3-OH	25
4-OH	61
5-OH	72
Total	169

Table S1: Summary of atomic configurations analyzed. [a] Unique non-dissociated configurations identified after DFT relaxation. [b] Partial estimate considering only top site occupancy of OH.

2. Atomic and bond attributes used to generate the crystal subgraph network motifs

Every subgraph, with nodes representing atoms and edges representing bonds, is converted to a featurized object composed of separate node features and edge features (see Methods section for additional details). Node features are binary one-hot encodings of an element's chemical and geometric attributes. Meanwhile, the edge features encode the spatial bond length for each node pair. Finally, every surface representation has a corresponding target property which is attached to the graph object. In this case, we have used the average binding energy of adsorbates on the surface.

System	Attribute	Range	# of interval
Atom	Pauling electronegativity	0.5 - 4.0	10
	Cordero covalent radius [pm]	25 - 250	10
	Valence electrons	1-12	12
	First ionization energy [eV][a]	1.3 – 3.3	10
	Co-ordination number	0 - 12	12
Bonds	Bond length [Angstrom][b]	0.5 – 3.5	6
State	Average binding energy	-	-

Table S2: Atomic and bond attributes used to generate the crystal subgraph network motifs. [a]Log scale is used to encode this property. [b]One-hot encoding (this work) or Gaussian basis (elsewhere[1]) can be used.

3. Model architecture

The forward pass for the ACE-GCN, once subgraphs are generated for high coverage adsorbate configurations, is done by applying graph convolutions on the individual sub-graphs, as implemented in previous accounts, [1,2] where node features $x_v^{(l)}$ of all nodes $v \in V$ in a graph $G = (V, E)$ are iteratively updated by aggregating localized information from their neighbors $N(v)$.

Training a GNN for global graph regression is performed as follows:

1. Embed each node by performing multiple rounds of graph convolutions
2. Aggregate node embeddings into a single graph embedding (readout layer)
3. Train a fully-connected neural network on the graph embedding with output as the target property of choice

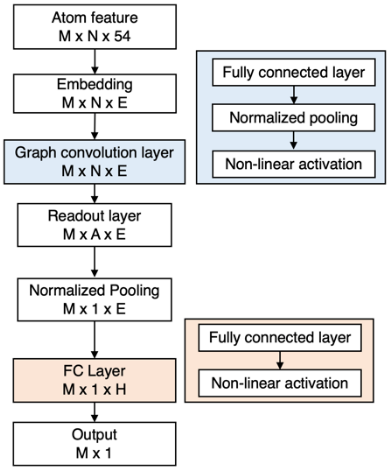

Figure S1: Schematic of the forward pass in the ACE-GCN model. Each atom feature and corresponding edge attributes are operated.

a. Pooling operation through supplemental indexing

Hierarchical pooling operations are employed in the ACE-GCN model to allow encoding of arbitrary sized subgraphs. As such, the model can account for a variable number of neighbors, coverages, and different types of binding sites on the catalyst surface. Every subgraph, node, and neighbor are labeled by a supplemental indexing scheme, as shown in the figure. Thus, when pooling the neighbors, the node index entry can be used to combine the features. A similar pooling scheme is extended to node properties and finally to the subgraphs themselves. The pooling operations are performed through PyTorch Geometric's Scatter method (https://pytorch-scatter.readthedocs.io/en/latest/). Through this method, elements in the input matrix of known dimensions can be reduced (via summation or mean operation) by explicitly specifying the indices which must be used for the said reduction. As a result, through successive hierarchical pooling, every high coverage configuration entry which is encoded as multiple subgraphs is condensed to a single fingerprint vector.

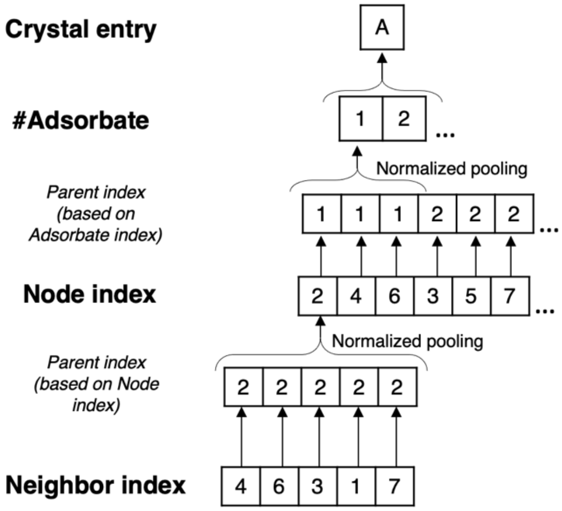

Figure S2: Hierarchical pooling scheme used in the ACE-GCN model. The pooling scheme used to make the final vector representation on which the ACE-GCN model is trained.

b. Model hyperparameters

Table S3 lists various model hyperparameters considered when constructing the ACE-GCN model. Besides the GCN module, the type of graph object and node encoding being developed can also be varied. The spatial bond attribute can be either expressed as simple one-hot encoding (as done in our case) or as Gaussian-based feature expansion (done in previous accounts [1]). We found that one-hot encoding worked best in our case since we seek to use the initial optimized configuration for predicting the stability of the converged high coverage adsorbates (see below for additional discussions). One hot encoding is insensitive to small fluctuations arising in the bond length from DFT relaxations (Refer Figure S3), whereas Gaussian-based featurization can be sensitive to changes in bond length, causing the resulting bond feature, during graph object generation, to vary significantly between relaxed and unrelaxed structures. These attributes are explained in detail in the next section. In summary, it is possible to modulate the model's sensitivity to the bond features using the two featurization techniques.

Appropriate set of hyperparameters for NO^*/Pt_3Sn and OH*/Pt analysis were chosen through a grid search approach, wherein multiple set of ACE-GCN model parameters were considered and the set which provided lowest mean absolute error (MAE) for the validation set were chosen. As a starting point for the model optimization, hyperparameters used by Xie et. al. were selected. Overall we observed that the model performance was most affected by the learning rate while other hyperparameters had less pronounced effect on the MAE. During the pretraining (incremental) of higher coverage configurations the same model employed in lower coverage were used.

System	Hyperparameters	Description
Bond encoding	One-hot encoding parameters:<br>- max/min bond length<br>- number of intervals	Encode spatial bond length as a one-hot encoding edge property
	Gaussian basis function:<br>- max/min bond length<br>- distance increments<br>- variance for the Gaussian distribution	Encode spatial bond length as a Gaussian basis
ACE-GCN model	Node embedding	Embedded node feature vector dimension
	Number of convolution layers	Determines the rounds of graph convolutions happening for a given subgraph
	Number of hidden layers	Number of hidden layers post message passing before the target property
	Length of each hidden layer	Number of nodes in each hidden layer
	Learning rate	Stochastic gradient descent learning rate
	Batch size	Mini-batch size for the stochastic gradient descent
	Dropout	Dropout for regularization and estimating uncertainty. Applied to fully-connected part

Table S3(a): List of ACE-GCN hyperparameters. List of hyperparameters used for bond encoding and training the ACE-GCN model.

System	Hyperparameters	NO*/PtSn	OH*/Pt
Bond encoding	One-hot encoding parameters	Min/Max: 0.5 – 3.5<br>Interval : 6	Min/Max: 0.5 – 3.5<br>Interval : 6
	Gaussian basis function	--	--
ACE-GCN model	Node embedding	20	30
	Number of convolution layers	5	6
	Number of hidden layers	2	3
	Length of each hidden layer	10	25
	Log learning rate	-7	-7
	Batch size	35	20
	Epochs	200	200
	Dropout	0	0

Table S3(b): ACE-GCN hyperparameters used in the analysis. The parameters are used as identified through grid search across parameter space.

c. Spatial bond attribute encoding

i. **Effect of type of spatial bond attribute encoding.** (A) and (B) plot the encoding vector generated for one-hot encoding and the Gaussian-feature expansion for a bond length. Considering 1.2 and 1.4 Å as examples, the bond feature for one-hot encoding will be the same, resulting in the same encoding in both cases. However, for the Gaussian-based encoding, there is a distribution of non-zero values, corresponding to the likelihood of that bond length being from Gaussian distributions that are generated in the user-defined range. Hence the two bonds, varying slightly in length, will be encoded differently. This sensitivity can be modulated by tuning the parameters generating the encodings, like the number of intervals, the mean, and the standard deviation of the Gaussian kernel employed. Nevertheless, there will always be some value associated with the bond features in the vicinity of the actual bond length value. This sensitivity, while useful for encoding the differences in the length, can make generalization of the model (trained on relaxed structures) to unrelaxed configurations difficult. As discussed in the next section, small deviations in the edge property may result in ACE-GCN network associating it with large energetic changes, since those are very different from the training set bond features. To mitigate such a response, one-hot encoding is chosen.

For the reasons discussed above, use of a Gaussian feature expansion could result in an ACE-GCN model which is highly sensitive to bond distances. This is not necessarily required when screening many different configurations where the major variations are expected to result from differences in binding environments (neighboring nodes) rather than spatial bond lengths. The one-hot encoding helps in modulating the model's sensitivity to such effects so that less emphasis is placed on the fluctuations in the bond distances, thus permitting systematic screening of high coverage configurations using initial optimized structures as guess atom positions.

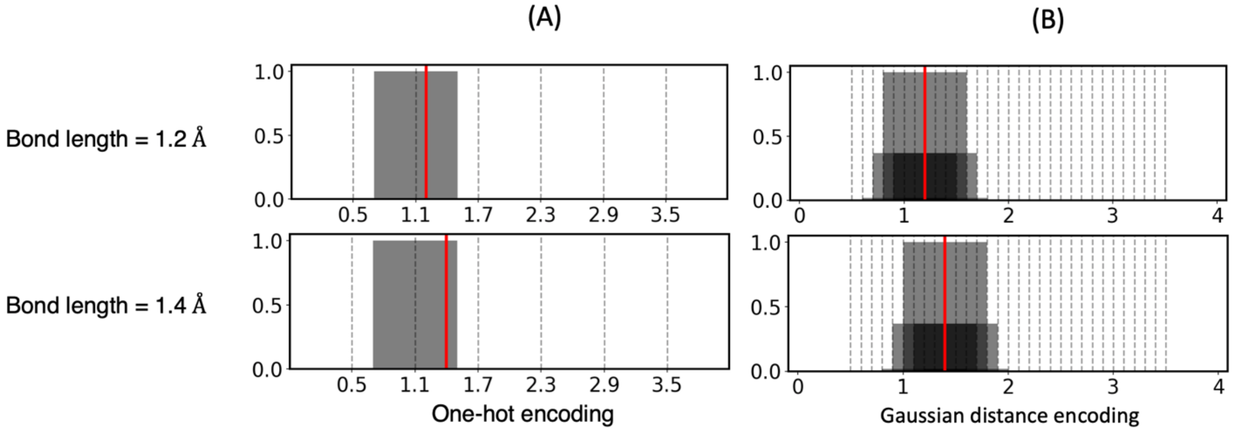

Figure S3: Comparison of bond features. The bond features are generated through (A) one-hot encoding and (B) Gaussian distance expansion. The red vertical line shows the bond length that is encoded at 1.2 Å

for the top and 1.4 Å for the bottom schematic. The black dashed lines show the bins generated for (A) one-hot and (B) Gaussian distance, respectively. The black shaded region shows the value of the encoding as the bond length is expressed either as (A) one-hot or as (B) Gaussian distance basis.

ii. **The effect of spatial bond encoding on the ACE-GCN model predictions.** The boxplot (Figure S4) shows the predicted energy difference for a given configuration between its unrelaxed state and post-DFT relaxation. The plot to the left is the generated by encoding bond distances as one-hot encodings, whereas the energy difference in the right was plotted for cases where bond distances were encoded as Gaussian window (refer Figure S3 for more details). All other model hyperparameters were kept the same during the network training. The energy difference (y-axis), as predicted by ACE-GCN for relaxed and unrelaxed cases, are plotted as a function of the net coordination number difference (x-axis). It is seen that the range, given by spines of the box plot, is larger for the predictions with Gaussian encodings compared to one-hot. The inter-quartile range is also larger for the Gaussian encodings, suggesting more variance. The table below each box plot lists the key statistics for the energy difference (y-axis of the box plot) estimated for each net coordination difference (x-axis of box plot). In particular, for cases where binding sites did not change post DFT-relaxation (given by coordination difference of 0), the mean and standard deviation for Gaussian window encoding is larger than that for one-hot encoding. Similar conclusions can be drawn for those belonging to other cases (refer to the mean and standard deviation column entries in Figure S4).

Ideally, to systematically sample low energy configurations, an appropriate initial guess for a strong binding adsorbate, such as NO* on $Pt_3Sn(111)$, should be sufficient for choosing candidates for subsequent, expensive DFT relaxation. To facilitate this type of analysis in the ACE-GCN formalism, we use an encoding which is more sensitive to the chemical geometric environment of each atom in the graph than to the intrinsic bond length of every atom pair (edge property of pairwise nodes in the subgraph). Doing so allows us to propose unrelaxed structures which often result in stable configurations after relaxation. Since the model is trained on converged configurations, such an approach ensures that the model energy predictions are not overly sensitive to the bond distances, which would make predictions using initial configurations more difficult. In general, while either approach could be used with ACE-GCN, we conclude, on balance, that the one-hot method is more useful for our intended purposes.

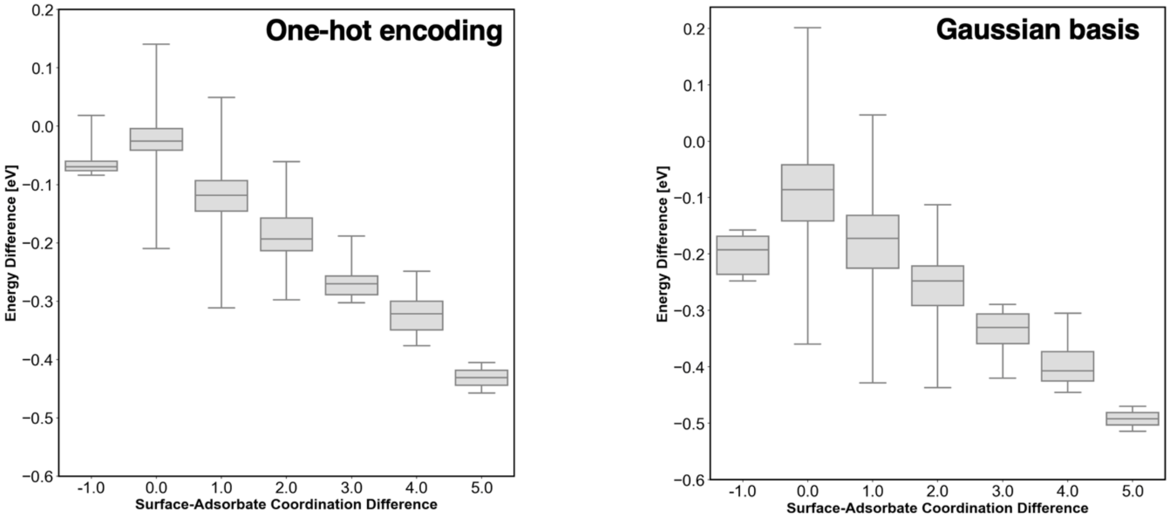

Coordination difference	Energy difference between relaxed and unrelaxed configurations [eV]			
	Mean	Standard Deviation	Min	Max
-1	-0.057	0.038	-0.084	0.018
0	-0.028	0.045	-0.210	0.140
1	-0.119	0.049	-0.312	0.050
2	-0.187	0.043	-0.298	-0.061
3	-0.265	0.034	-0.303	-0.188
4	-0.321	0.044	-0.376	-0.249
5	-0.431	0.036	-0.457	-0.406

Coordination difference	Energy difference between relaxed and unrelaxed configurations [eV]			
	Mean	Standard Deviation	Min	Max
-1	-0.201	0.040	-0.248	-0.158
0	-0.093	0.083	-0.360	0.202
1	-0.179	0.075	-0.429	0.047
2	-0.255	0.061	-0.437	-0.113
3	-0.336	0.039	-0.420	-0.289
4	-0.394	0.048	-0.446	-0.305
5	-0.492	0.031	-0.514	-0.470

Figure S4: Effect of different encodings on ACE-GCN model prediction. Box plot representing the effect of bond edge encoding on the energy prediction by ACE-GCN for relaxed and unrelaxed 4NO* configurations on Pt_3Sn(111). For the same set of 4-NO* configurations, the ACE-GCN model was used to predict the energy for unrelaxed and DFT-relaxed configurations. The degree of reconstruction metric is determined by considering the change in net coordination of the NO molecules on the Pt_3Sn(111) slab pre- and post-DFT relaxation. The net coordination is estimated by adding the coordination of all the NO molecules, with top binding considered as 1, bridge as 2, and threefold (fcc/hcp sites) as 3. The height of each box represents the interquartile range, i.e. the value corresponding to the 25th and 75th percentile. The line in the middle of each box is the median of the sample. The spines drawn out of the top and bottom show the minimum and maximum energy difference observed.

4. NO/Pt_3Sn(111) Analysis

a. Screening high coverage configurations using the initial (unrelaxed) structures

Figure S5 compares average NO* binding energy predictions from ACE-GCN (x-axis) with the DFT energies of the corresponding (fully relaxed) configurations plotted on the y-axis (see Methods for details of the calculations). All NO* configurations identified in the analysis are plotted in the diagram. Statistics for each coverage are listed in Table S4. A few of the configurations predicted to be unstable, as per initial (unrelaxed) guesses, relax to stable arrangements after DFT optimization. Such a relaxation is attributed to the shifting of NO binding location. The table below each plot describes the statistics for the given NO* coverage. The ACE-GCN model can be used to predict binding energies on the unoptimized geometries or the optimized geometries. The latter comparison is

accurate and closely follows the DFT predictions, as the structures of both configurations are very similar. Looking at the error (ΔBE_{NO}) column, the error for the DFT/optimized column has mean of 0.01 eV for 4/5/6-NO* coverage. Parity plots for comparing the ACE-GCN predicted energies and DFT-optimized energies are discussed in subsequent sections.

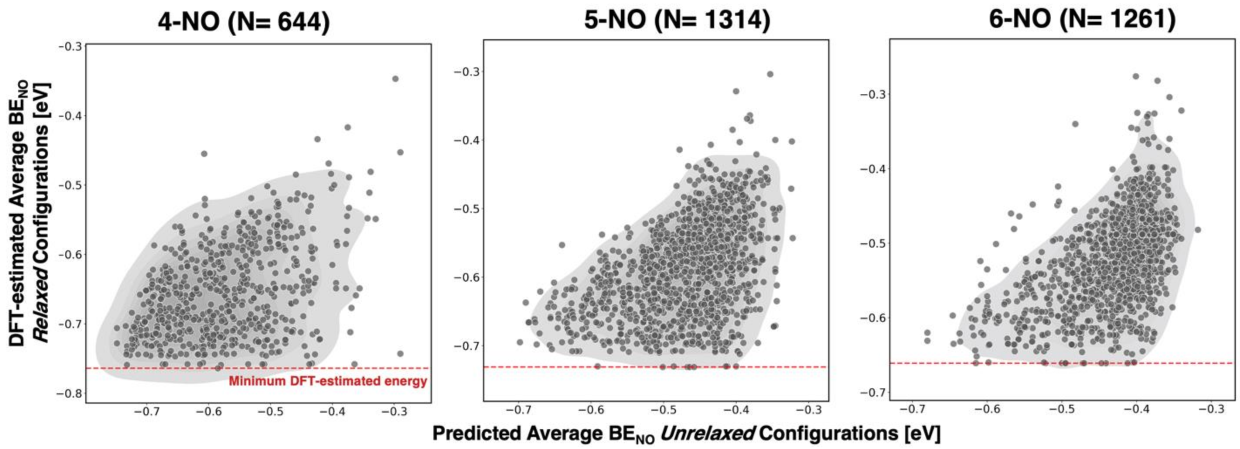

Figure S5: Plot of ACE-GCN predicted BE_{NO} and DFT-optimized BE_{NO} energies. ACE-GCN model showing the inclusion of all the data points, including the structures that significantly relaxed during DFT. See main text for descriptions of how the algorithm was retrained for each higher coverage level.

(a)

4-NO	ACE-GCN Predicted BE_{NO}		BE_{NO} (DFT)	Difference in BE_{NO} estimates (ΔBE_{NO})		
	on Unrelaxed	on Relaxed		DFT - Unrelaxed	DFT - Relaxed	Unrelaxed - Relaxed
**Mean**	-0.58	-0.67	-0.65	-0.08	0.01	-0.09
**Std**	0.09	0.08	0.07	0.09	0.05	0.08
**Min**	-0.75	-0.83	-0.76	-0.45	-0.20	-0.46
**Max**	-0.29	-0.30	-0.29	0.15	0.18	0.14

(b)

5-NO	ACE-GCN Predicted BE_{NO}		BE_{NO} (DFT)	Difference in BE_{NO} estimates (ΔBE_{NO})		
	on Unrelaxed	on Relaxed		DFT - Unrelaxed	DFT - Relaxed	Unrelaxed - Relaxed
**Mean**	-0.47	-0.58	-0.58	-0.12	0.00	-0.12
**Std**	0.07	0.07	0.07	0.07	0.04	0.08
**Min**	-0.69	-0.76	-0.73	-0.33	-0.20	-0.37
**Max**	-0.32	-0.32	-0.30	0.08	0.17	0.07

(c)

6-NO	ACE-GCN Predicted BE_{NO}		BE_{NO} (DFT)	Difference in BE_{NO} estimates (ΔBE_{NO})		
	on Unrelaxed	on Relaxed		DFT - Unrelaxed	DFT - Relaxed	Unrelaxed - Relaxed
**Mean**	-0.43	-0.54	-0.52	-0.09	0.02	-0.11
**Std**	0.06	0.06	0.06	0.06	0.03	0.06
**Min**	-0.68	-0.72	-0.66	-0.26	-0.12	-0.33
**Max**	-0.32	-0.39	-0.28	0.14	0.19	0.11

Table S4: Summary statistics comparing the average BE_{NO} estimated through ACE-GCN on unrelaxed and DFT-relaxed structures to the BE_{NO} value estimated from DFT for all of the (a) 4-NO, (b) 5-NO, and (c) 6-NO configurations. ACE-GCN predicted BE_{NO} and DFT columns lists the raw values of the binding energy computation. The ΔBE_{NO} column lists the pairwise energy difference between types of energy estimates (the error, if ACE-GCN predicted energies on DFT-relaxed configurations are compared to DFT values). It is observed that the error in predicting BE_{NO} for relaxed structures and DFT estimates is very low (DFT – relaxed column), suggesting that the mapping of geometric fingerprints to energies is robust.

b. Degree of reconstruction analysis for NO*

Figures S6 presents box plots comparing the ACE-GCN predicted average binding energies (A), the corresponding energy differences post-DFT relaxation (B) for the high coverage NO* configurations on the y-axis, and the degree of reconstruction of the configurations after DFT relaxation on the x-axis. The degree of reconstruction metric is determined by considering the change in net coordination of the NO molecules on the $Pt_3Sn(111)$ slab pre- and post-DFT relaxation. The net coordination is calculated by adding the coordination of all the NO* adsorbates, with top-site binding considered as 1, bridge as 2, and three-hold (fcc/hcp sites) as 3. The energy range (A) corresponding to a coordination difference of 0.0 represents the ACE-GCN energy predictions for all the configurations initially enumerated by SurfGraph. No other coordination occupies the same span as the 0.0 sample. In turn, the median of the energy difference for configurations post-DFT relaxation is 0.00 eV for cases where the net coordination change is 0. Interestingly, the median for energy difference increases, that is, structures relax considerably more, when the initial arrangement is unstable. Hence, the representations predicted to be unstable by ACE-GCN undergo most relaxation and show the highest energy change.

The results demonstrate that for all coverages considered, the initial configurations enumerated by SurfGraph span all the ACE-GCN energies associated with the DFT-relaxed configurations. This suggests that we do not lose information when we consider only the configurations which have not changed in terms of overall NO* coordination during DFT relaxation, as plotted in Figure 3 (main text).

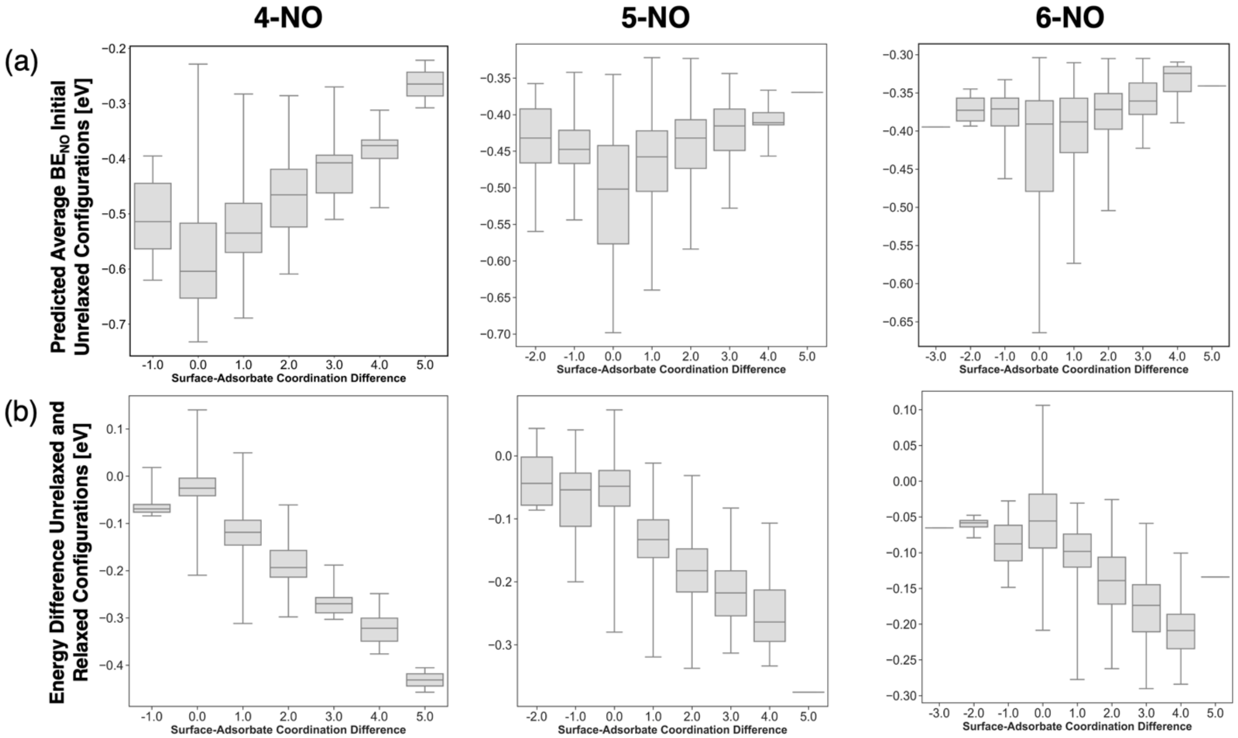

Figure S6: Analysis of the degree of surface reconstruction (surface-adsorbate coordination difference). Box plots indicating the (a) ACE-GCN predicted BE_{NO} on unrelaxed structures, as generated by SurfGraph vs. adsorbate coordination difference, and (b) Energy difference of ACE-GCN predicted BE_{NO} between unrelaxed and DFT-relaxed structures vs. net difference in adsorbate coordination before and

after DFT relaxation. The adsorbate coordination difference is calculated by determining the summation of total coordination of NO binding sites before and after DFT relaxation. Each box in the plot represents the interquartile range from the 25th to the 75th percentile of the data. The line in the middle of each box is the median of the sample. The spines drawn out of the top and bottom of the box terminate at the minimum and maximum values of the sample.

c. Representative stable/unstable configurations identified by ACE-GCN

To showcase the non-linear mapping, from geometric fingerprints to target, being developed by ACE-GCN in the NO* energy predictions, selected configurations for 4/6-NO* coverages are shown in Figure S7 with corresponding energetics listed in Table S5. The site difference column in Table S5 denotes the change in net NO* coordination number before and after DFT relaxation (see discussion above).

In the high energy (unstable) case, as observed from in our previous analysis, [3] NO* molecules are primarily bound to the bridge sites, with few occupying threefold sites. For the low energy (stable) cases, top site occupancy is found to be very common. This is true for both the 4-NO* and 6-NO* cases and is predicted independently by ACE-GCN with no need for further DFT optimization. Finally, in cases where structures generated by SurfGraph undergo considerable reconstruction, the energies in the DFT-relaxed structures (both DFT and ACE-GCN) are, not surprisingly, quite different from the initial energies. *In all cases, if given a DFT-relaxed configuration, ACE-GCN predicts average NO binding energies that are close to the DFT-relaxed energy.*

Types of NO* / Pt3Sn Configuration		ACE-GCN prediction [eV]		DFT [eV]	Net NO* coordination difference
		Unrelaxed	Relaxed	Relaxed	
**4-NO / Pt_3Sn**					
1	**Unstable**	-0.30	-0.30	-0.34	0.0
2	**Stable**	-0.74	-0.76	-0.71	0.0
3	**NO* binding site change**	-0.29	-0.74	-0.74	5.0
**6-NO / Pt_3Sn**					
1	**Unstable**	-0.37	-0.37	-0.39	0.0
2	**Stable**	-0.68	-0.68	-0.66	0.0
3	**NO* binding site change**	-0.39	-0.67	-0.64	4.0

Table S5. DFT-estimated and ACE-GCN-predicted (on relaxed and unrelaxed configurations) BE_{NO}. For configurations with no reconstruction, the net NO* coordination difference columns is 0.0, and the model captures the energetics quite well. In all cases, ACE-GCN correctly predicts DFT energies if given the final, DFT-relaxed structures.

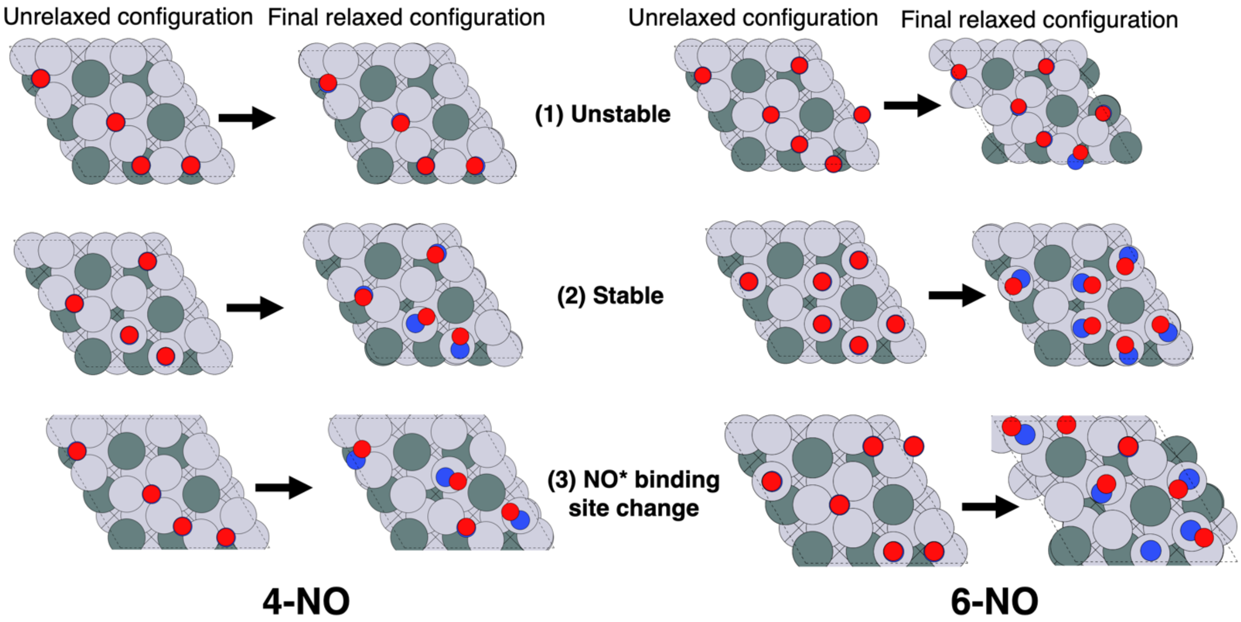

Figure S7: Different types of configurations in the dataset. Schematics of 4/6-NO* configurations corresponding to the configurations listed in Table S5. A single periodic unit cell is depicted in each case.

d. Parity plots for training of ACE-GCN model

The ACE-GCN model performance when trained on the 123-NO* dataset is shown in Figure S8(a). The performance is improved when some portion of the 4-NO* points (the 100 most and least stable configurations, as ranked by ACE-GCN) are incorporated in the model training and validation (Figure S8(b)).The 4-NO* dataset, comprising 200 points, is split into training and testing sets in an 80-20 ratio. The testing set (40 points) is kept aside for testing ACE-GCN model performance. The training set (160 points) is included in the model training and validation in (b) where the ACE-GCN model is trained / validated on the combined dataset of 1/2/3-NO* + 4NO* points.

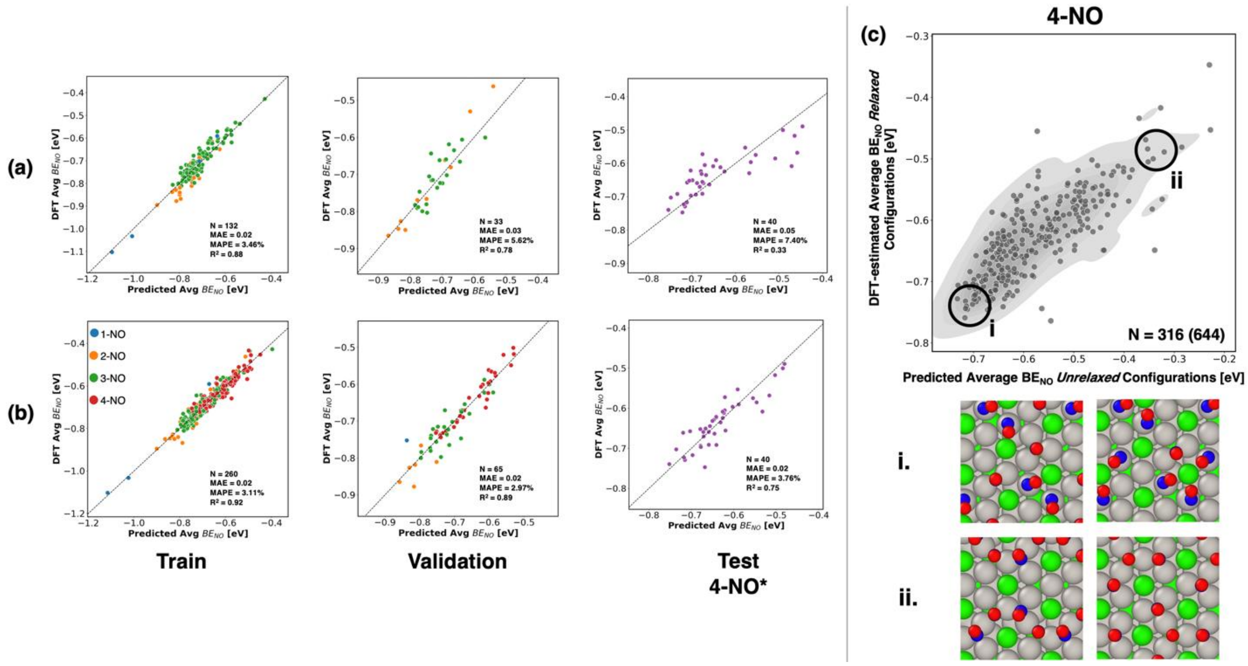

Figure S8: Parity plots for training, validation, and test sets. Plots for (a) 1/2/3NO* and (b) incremental 1/2/3/4 NO* ACE-GCN model. For consistency, the test set is generated from 20% of selected 4-NO* and used for evaluating model performance in (a) and (b). The panel (c) shows the prediction of the ACE-GCN model trained on 1/2/3-NO* for 4-NO* configurations, only those configurations where NO* binding sites didn't change after relaxation are plotted. All configurations are plotted in Figure S5. This analysis is as per discussion in Figure 3, main text. (i) and (ii) highlight the region of low (and high) energy in the plots and the representative NO* configurations observed post-DFT relaxation.

Figure S9 depicts ACE-GCN performance using an incremental training strategy, wherein a limited number of DFT-relaxed energies at given NO* coverages are systematically added to the training set and used to predict energies on the DFT-relaxed configurations for the subsequent coverage level. All of the 1/2/3 NO* configurations are included in the model training, but only 80% of total possible 4/5/6 NO* configurations are considered in the network training exercise. The remaining 20% are held out for testing purposes (hold-out test set). The performance of the ACE-GCN model on the held-out test set of 5/6 NO* is plotted below each training set parity curve. Adding more DFT-relaxed energies to the training sets, of course, slightly increases the accuracy of the ACE-GCN predictions, but at the price of reduced computational efficiency.

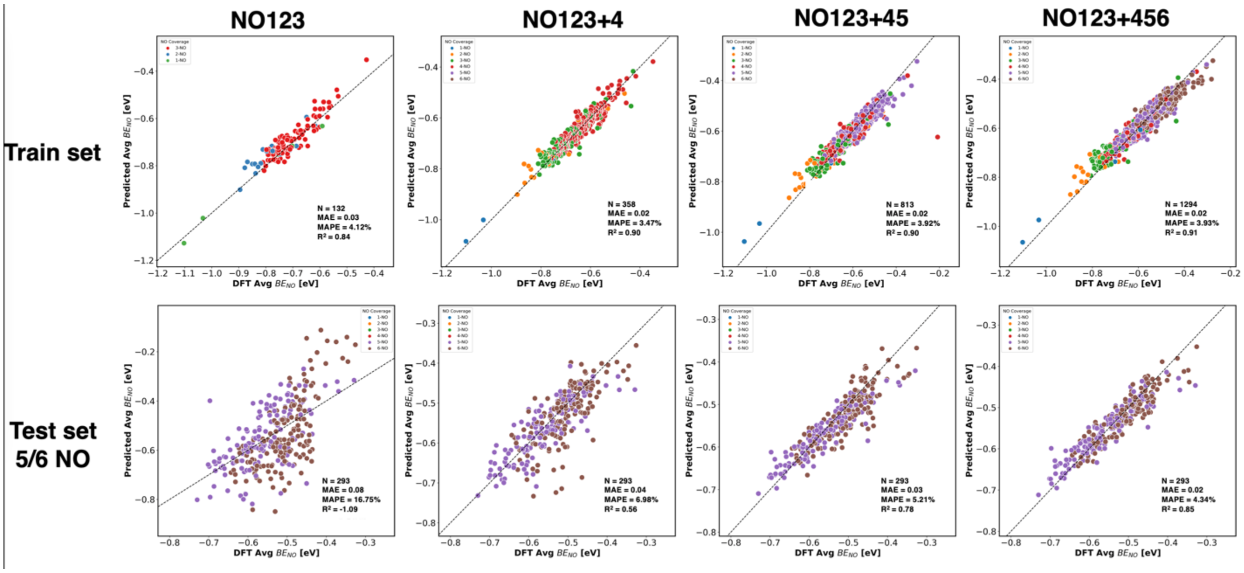

Figure S9: Parity plots for incremental training strategy incorporating higher coverage DFT-relaxed NO* configurations in the ACE-GCN model training. Starting from left, the ACE-GCN model is training on increasing NO* coverage configurations, and the trained model is tested every time on the same set 5/6 NO* configurations (hold-out test set). In the model training shown here, the ACE-GCN is trained with a train/validation set of 80/20 on the input data.

e. MAPE of the ACE-GCN model as a function of number of high NO* coverage configurations included in the model training

In this analysis, we consider all NO* configurations and test the ACE-GCN model prediction error as a function of different training data portions. All 1,2,3-NO* points are used in the training set. For 4/5/6-NO* data, the dataset is randomly split in a 80-20 ratio. This way, the 80% of the data (hold-out training) is gradually incorporated in the model training, and the remaining 20% of data (hold-out testing) is reserved for estimating the model performance (e.g. estimating the MAPE, Figure S10, using only the test data). As shown in Figure S10, considering the 4-NO* case, gradually introducing more data, out of the 80% reserved dataset, increases the ACE-GCN performance for predicting 4-NO points. The performance plateaus when around 50% of the hold-out training data is included in the model training. A similar analysis is conducted for 5/6-NO*.

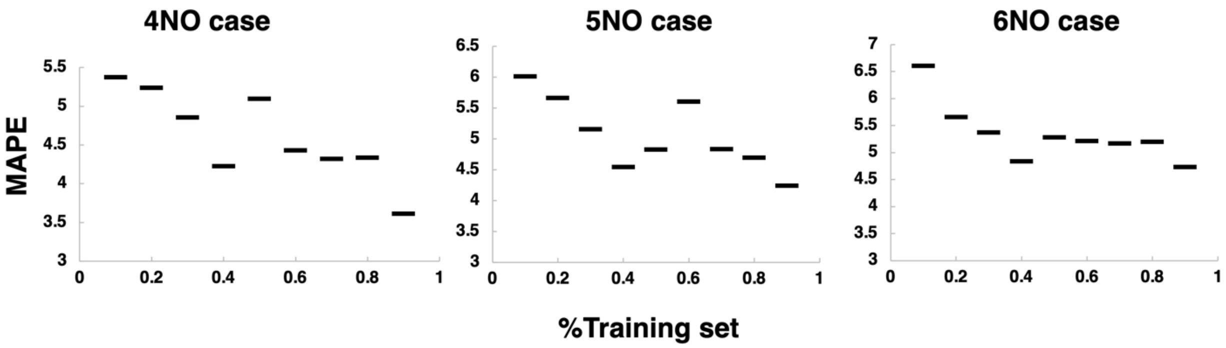

Figure S10: MAPE of the ACE-GCN model as a function of number of high NO* coverage configurations included in the model training. Mean absolute percentage error (MAPE) for the testing of 4/5/6-NO* cases as a function of data size. The x-axis shows the ratio of training cases of 4/5/6-NO*

being included in the model training. It is evident that incorporating more points corresponding to a given coverage reduces the model prediction error.

f. 5-fold cross validation of the ACE-GCN model

Table S6 shows the mean absolute percentage error (MAPE), mean absolute error (MAE), and root mean square error (RMSE) for test datasets comprised of varying NO* coverages. Cross-validation is done using the K-fold (K=5) CV strategy. Before training/validation of the model, all the 4/5/6-NO points are split in a 80-20 ratio. 80% are reserved for training and 20% are reserved for testing (hold-out testing set). The fitting results in Table S6 are reported as per the model's performance on the hold-out test set. The splits are kept the same for consistency. Based on the coverage being analyzed, the final dataset is split in K number of folds for cross validation. Finally, model testing for each fold is performed on the hold-out test-set that is kept aside. The result provided in the table is the average, with 1 standard deviation error bars, for the 5-fold runs. Training/validation for 4-NO points is carried out using NO-123 points with an 80-20 split. For 5/6-NO points training/validation is carried on all NO-123 points and 80% of 4-NO points.

Test set	MAPE	MAE [eV]	RMSE [eV]
NO/Pt_3Sn			
**4NO**	6.82 ± 1.37	0.03 ± 0.01	0.03 ± 0.01
**5NO**	6.19 ± 0.77	0.03 ± 0.01	0.04 ± 0.01
**6NO**	8.40 ± 1.09	0.04 ± 0.01	0.05 ± 0.01

Table S6. Table listing the 5-fold cross-validation ACE-GCN errors for the 4/5/6-NO* dataset. MAPE = Mean absolute percent error, MAE = mean absolute error, RMSE = root mean square error, all reported in eV.

5. OH/Pt(100) and Pt(221) Analysis

Screening high coverage OH* configurations on Pt(221)

Figure S11 shows all configurations for 4/5/6 OH* that were selected for DFT optimization. Some of these configurations resulted in OH bonds being dissociated (marked by light blue points). Although these configurations have low DFT-relaxed binding energies, they will likely be less stable if water solvation effects are considered, and the ACE-GCN algorithm is not configured to rigorously analyze such structures. Hence, these configurations are not considered in the model training. For each OH* coverage, SurfGraph is used to create the initial configurations. The ACE-GCN model then predicts the average BE for those configurations. DFT relaxation is carried out on candidates selected from the most stable and unstable regions to rigorously test the accuracy of the ACE-GCN model.

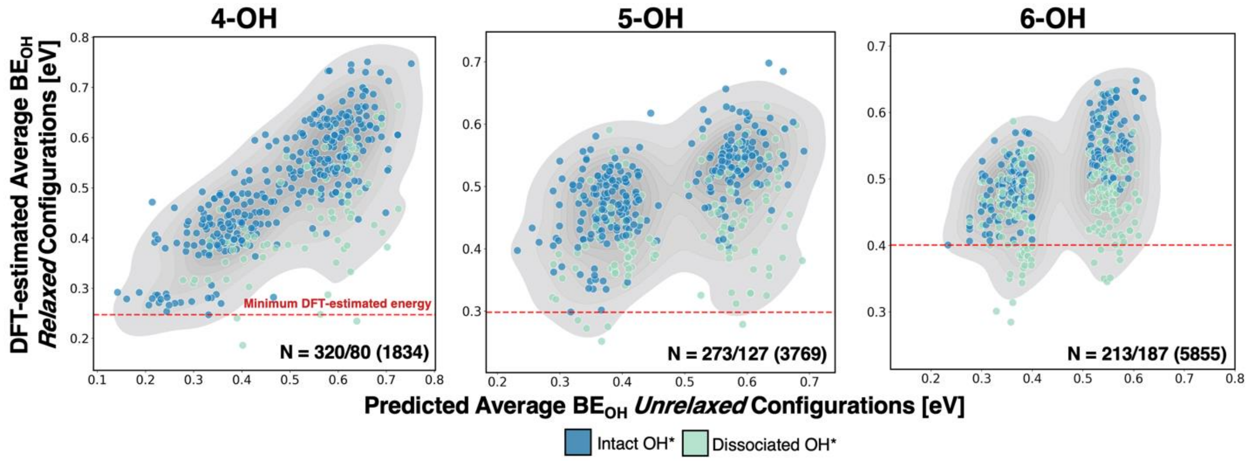

Figure S11. Scatter plots for predicted and DFT-relaxed average OH* binding energies on Pt(221) for all 4, 5, and 6-OH* configurations identified by SurfGraph and ranked by ACE-GCN. The dark blue points show the configurations where all the OH* are intact, and light blue points indicate the cases where at least one OH* is dissociated. For each coverage, the most and least stable 200 candidates (400 total) are chosen for DFT relaxation according to the ranking of the configurations by ACE-GCN. The number in the inset shows the ratio of number of configurations that had intact OH* to the number that contained dissociated OH* (out of 400 cases), with total possible enumerations mentioned in the bracket. The analysis presented here is equivalent to that presented in Figure 3 (main text), although here dissociated configurations are plotted, as well.

Influence of different OH* training sets on 4-OH* prediction

Figure S12 explores the effect of using different datasets for training the ACE-GCN model for ranking 4-OH* on Pt(221) configurations. Energy post-DFT relaxation is used for validating the ranking. In all cases, the same set of 400 points of 4-OH* (as discussed in the previous section and the main text) are used for ACE-GCN energy predictions (shown on the x-axis), while the y-axis is the DFT-relaxed energy. A) Only Pt(100) OH points are used for training ACE-GCN model, which is then used to predict energies for the 400 4-OH* Pt(221) cases. Here, the ACE-GCN predicted initial energy does not correlate with the final energy. B) Only 1,2,3-OH* on Pt(221) configurations are used in the model training. There is improvement in the ranking of 4-OH* structures. C) The combined dataset of Pt(100) and Pt(221) points is used for training, and this ACE-GCN is used to rank the 4-OH* on Pt(221). This model shows further improvement in ranking the 4-OH* configurations, especially in the low energy (stable) region.

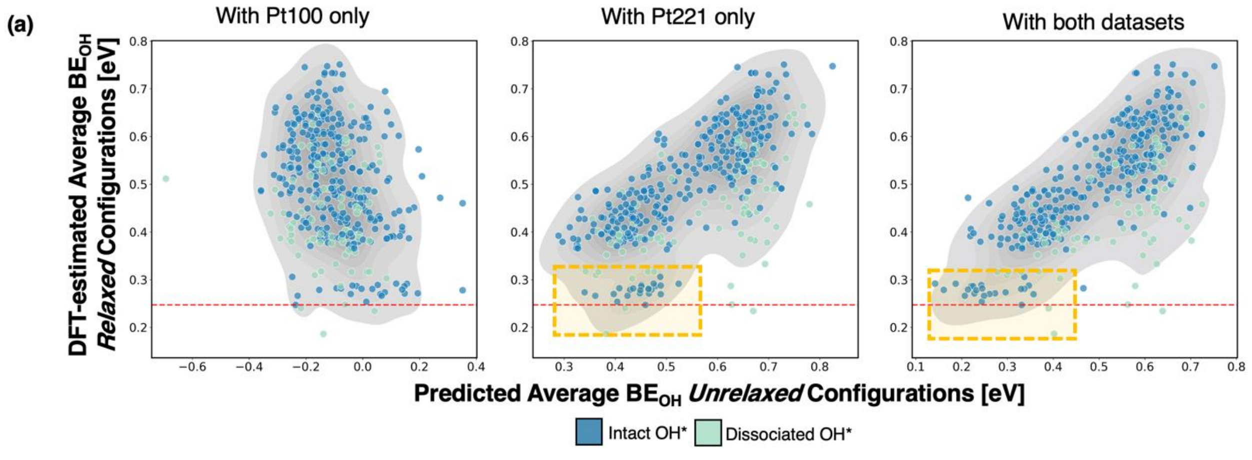

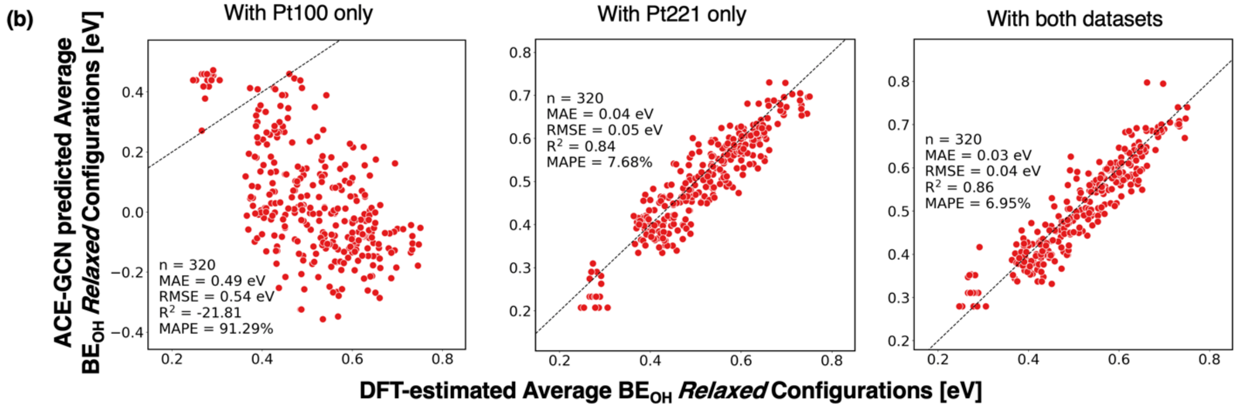

Figure S12: Analysis with different datasets for OH* adsorption. (a) Scatter plot depicting the improvement in 4OH* ranking of the cases as a function of different OH training points. The orange regions in the middle and right scatter plots show the specific improvement in model prediction. (b) Parity plot showing the ACE-GCN model performance on predicting energy of selected DFT-relaxed 4-OH* configurations. The same set of 4NO* points are used for ACE-GCN energy prediction. Using both Pt(100) and Pt(221) datasets, in the right-most parity plot, yields slightly improved parities.

Figure S13 shows parity plots for the variation in the ACE-GCN model prediction performance as higher coverage (4/5 OH*) cases are incorporated in the model training. In all cases, a random 80-10-10 split to generate training, validation, and testing is used for network training and prediction. Starting from Figure S14 (left), the parity plot shows the performance of ACE-GCN in estimating the average binding energy of DFT-relaxed 4 and 5 OH* configurations with the ACE-GCN model trained only on energies and geometries of DFT-relaxed Pt(100) 1-5OH and Pt(221) 1/2/3 OH* points. Next, following the incremental training strategy described in Figure 1 (main text), the ACE-GCN model is retrained on selected non-dissociated DFT-relaxed 4-OH* points (320 cases). The model performance is shown in the middle parity plot for predicting energetics on DFT-relaxed 4/5 OH* points. Next, this model was retrained using the selected non-dissociated 5-OH* (273) cases and incorporated into the model training. The prediction error for 4/5OH* data points, especially 5OH*, is seen to decrease, given by lowering MAPE and increasing R^2 from left to right, as the model is sequentially trained on higher coverage cases.

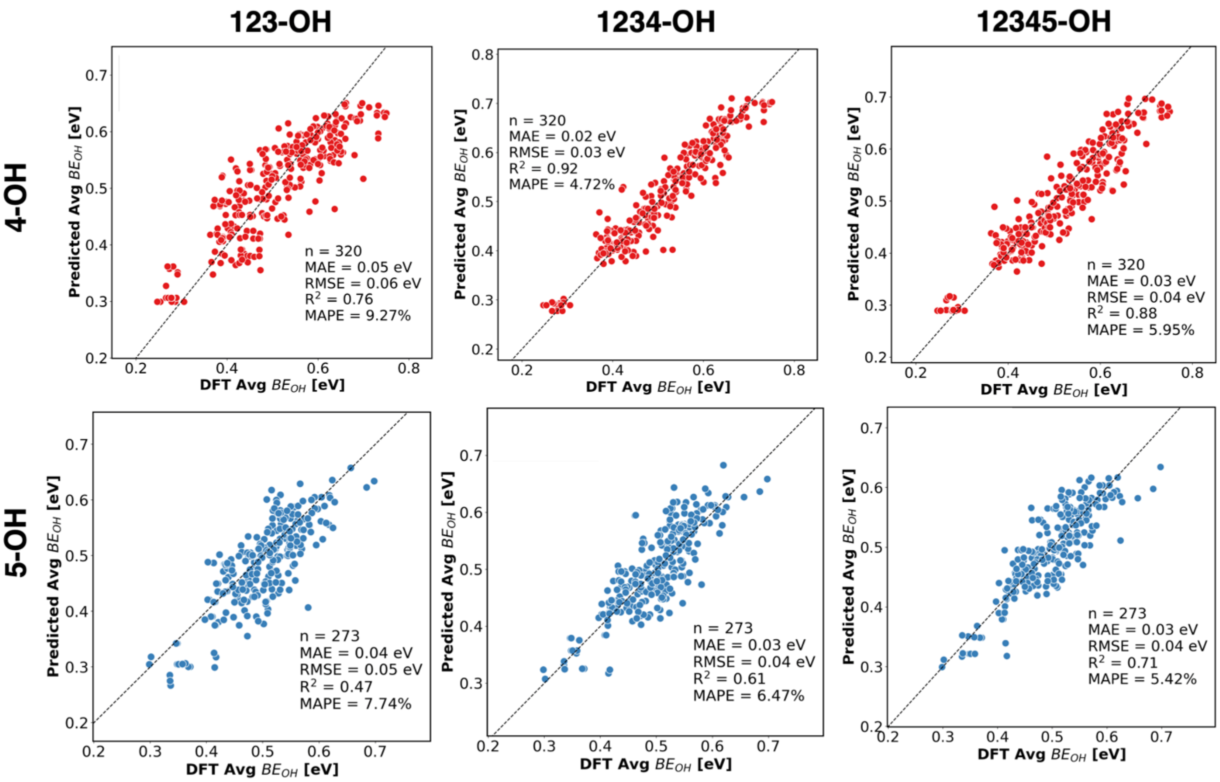

Figure S13: Incremental training for OH* adsorption. Incremental training strategy wherein DFT-relaxed, non-dissociated, high coverage configurations of 4/5 OH* are selected from the total set of identified cases, as discussed in the OH/Pt selection (main text).

Supplementary References

1. Xie, T. & Grossman, J. C. Crystal Graph Convolutional Neural Networks for an Accurate and Interpretable Prediction of Material Properties. *Physical Review Letters* **120**, 1929 (2018).

2. Gilmer, J., Schoenholz, S. S., Riley, P. F., Vinyals, O. & Dahl, G. E. Neural Message Passing for Quantum Chemistry. in 1263–1272 (PMLR, 2017).

3. Deshpande, S., Maxson, T. & Greeley, J. Graph theory approach to determine configurations of multidentate and high coverage adsorbates for heterogeneous catalysis. *npj Computational Materials* **6**, 4981 (2020).
